# Precision spherical nucleic acids for delivery of anticancer drugs†
†Electronic supplementary information (ESI) available: DNA nanoparticle design and assembly, evaluation of BKM120 encapsulation, structural characterization, stability and shelf-life of drug-loaded structures, cellular uptake in cancer cells, *in vitro* cell studies, HSA binding experiments and *in vivo* studies. See DOI: 10.1039/c7sc01619k



**DOI:** 10.1039/c7sc01619k

**Published:** 2017-07-05

**Authors:** Danny Bousmail, Lilian Amrein, Johans J. Fakhoury, Hassan H. Fakih, John C. C. Hsu, Lawrence Panasci, Hanadi F. Sleiman

**Affiliations:** a Department of Chemistry , Centre for Self-Assembled Chemical Structures (CSACS) , McGill University , 801 Sherbrooke St. W. , Montreal , Canada . Email: hanadi.sleiman@mcgill.ca; b Department of Oncology , Jewish General Hospital , 3755 Cote Sainte-Catherine Rd. , Montreal , Canada . Email: lpanasci@hotmail.com

## Abstract

We report a spherical nucleic acid (SNA) system for the delivery of BKM120, an anticancer drug for treatment of chronic lymphocytic leukemia (CLL). While promising for cancer treatment, this drug crosses the blood–brain barrier causing significant side-effects in patients. The DNA nanoparticle encapsulates BKM120 in high efficiency, and is unparalleled in its monodispersity, ease of synthesis and stability in different biological media and in serum. These DNA nanostructures demonstrate efficient uptake in human cervical cancer (HeLa) cells, and increased internalization of cargo. *In vitro* studies show that BKM120-loaded nanoparticles promote apoptosis in primary patient CLL lymphocytes, and act as sensitizers of other antitumor drugs, without causing non-specific inflammation. Evaluation of this drug delivery system *in vivo* shows long circulation times up to 24 hours, full body distribution, accumulation at tumor sites and minimal leakage through the blood–brain barrier. Our results demonstrate the great potential of these delivery vehicles as a general platform for chemotherapeutic drug delivery.

## Abstract 2

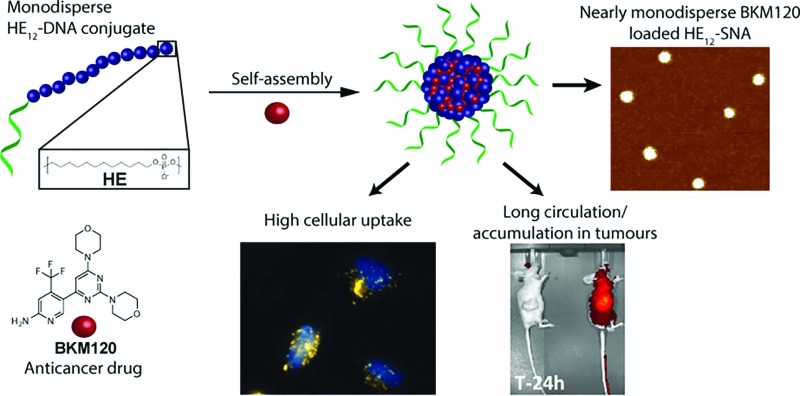
Highly monodisperse sequence-defined spherical nucleic acids (**HE_12_–SNAs**) for delivery of small-molecule anticancer drugs.

## Introduction

Targeted action of small-molecule drugs remains a challenge in medicine. This holds true for antitumor chemotherapeutic drugs, where much of their success has been hampered by off-target side-effects, poor pharmacokinetics and systemic toxicity.^1^ One effective approach to tackle this problem is the application of drug delivery systems that would protect the cargo along the administration route and direct it to its target site.^2^ Several delivery systems are currently being explored which include: dendrimers,^3^ liposomes,^4^ polymeric nanoparticles,^5^ micelles,^6^ protein nanoparticles,^7^ viral nanoparticles,^8^ inorganic nanoparticles^9^ and carbon nanotubes.^10^ However, many of them suffer from major limitations such as: toxicity, rapid clearance, complicated synthesis and particle heterogeneity.^11^ In particular, nanostructure size and shape have been demonstrated to play an important role in their biodistribution, circulation half-life, cellular targeting, efficacy and immune response.^12–15^ With most current drug delivery platforms suffering from structural polydispersity, the generation of monodisperse nanocarriers with well-defined structures will be essential for their application in drug delivery.

Polymers are the most commonly used material for developing nanoparticle-based drug carriers.^16^ In particular, amphiphilic block copolymers that contain a water-soluble block, and a hydrophobic block, have been extensively used as building blocks for chemotherapeutic drug delivery. These molecules phase-separate into micelles that contain a hydrophobic core which can accommodate lipophilic drug molecules and alter their kinetics both *in vitro* and *in vivo*.^17,18^ In recent years, a new class of amphiphilic block copolymers has also emerged which contains a hydrophobic synthetic polymer attached to a hydrophilic DNA segment, called DNA–polymer hybrids.^19^ These molecules can self-assemble into a wide-range of morphologies,^20^ including spherical micellar particles that expose a hydrophilic DNA shell and a hydrophobic core.^21,22^ A particularly successful example of such assemblies are spherical nucleic acids (SNAs).^23^ These structures are composed of a gold nanoparticle core and a corona of tightly packed DNA strands. SNAs have shown efficient cellular penetration and gene silencing ability both *in vitro* and *in vivo*.^24–26^

Recently, we reported a highly efficient and versatile method to generate DNA–polymer conjugates *via* solid-phase synthesis.^27^ Unlike conventional synthetic polymer chemistry, this method yields DNA–polymer conjugates that are fully monodisperse and sequence-defined. This class of material self-assembles spontaneously to generate highly monodisperse spherical micellar DNA nanoparticles in aqueous solution. Several examples in recent years have emerged demonstrating the suitability of DNA nanostructures in mimicking biological systems,^28,29^ construction of nanoelectronics^30^ and nanophotonics,^31^ and delivery of cancer therapeutics.^32^ Compared to DNA nanostructures, such as DNA origami,^33^ which require a large number of unique DNA strands to generate the designed structure,^32^ limiting their use in large-scale applications, micellar DNA nanoparticles are composed of only a single DNA–polymer conjugate strand. This type of hybrid strand also offers advantages over other block copolymers in that the DNA portion can be highly functional and programmable in the final structure.^34^ Additionally, the poly-phosphodiester units in both the oligonucleotide and hydrophobic portions of the DNA–polymer strands are biocompatible and biodegradable making them suitable for biological applications.^35^ Particles of self-assembled DNA–polymer conjugates expose a ssDNA corona, and have been used in ligand targeting,^22,36,37^ delivery of anti-sense oligonucleotides,^38–40^ DNA detection,^41^ formation of higher order assemblies,^42,43^ and templating organic reactions.^44,45^ In particular, these DNA particles have shown great potential in cancer therapy.^22,36,46^ However, the exploration of these structures for cancer therapy has only been limited to *in vitro* cell studies. Our interest also focuses on cancer therapy, specifically, the development of a DNA nanoparticle delivery system for BKM120, an anticancer drug towards the treatment of Chronic Lymphocytic Leukemia (CLL).

Chronic Lymphocytic Leukemia (CLL) remains the most common type of leukemia with an incidence rate of approx. 4/100 000 people in the United States.^47^ Current treatments of CLL include chemotherapeutic agents such as alkylating agents (chlorambucil, cyclophosphamide and bendamustine), purine analogs (fludarabine) and immunotherapeutics (Rituximab, Alemtuzumab).^48,49^ The current gold standard for treatment is through chemoimmunotherapy; a combination of fludarabine, cyclophosphamide and rituximab (FCR).^50–53^ Unfortunately, none of these treatments results in curative therapy providing strong justification for investigating new therapeutic approaches for CLL. The phosphoinositide 3-kinase (PI3K) pathway has been shown to be a critical component of CLL survival and proliferation.^54–56^ The expression of PI3K triggers downstream cellular events that inhibit cell death by inactivating pro-apoptotic proteins.^57–60^ This makes the selective inhibition of PI3K a promising approach for the treatment of CLL and a focus of many efforts to develop novel inhibitors targeting this pathway.

Buparlisib (codenamed BKM120) is one such example of a pyrimidine-derived selective pan class I PI3K inhibitor.^61^ This molecule has shown high selectivity and potency against class I PI3Ks.^62^ BKM120 has demonstrated high cytotoxicity in B-chronic lymphocytic leukemia cells *in vitro*, and significant antitumor activity in human tumor xenograft models.^62,63^ Currently, this drug is under clinical investigation in advanced solid tumor and CLL patients.^64^ However, BKM120 can cross the blood–brain barrier and inhibit PI3K in the central nervous system (CNS), inducing anxiety, low serotonin levels, schizophrenia, and hindering its success for translation into the market. Hence, a strategy to effectively deliver BKM120 to its intended biological target without deleterious side-effects in the CNS would be a major goal for therapy with this small-molecule drug.

In this article, we report the development of a DNA nanoparticle platform for the delivery of BKM120. The drug-loaded structures are unique in their monodispersity, can be readily prepared and are stable in different biological media and in serum. We show the increased cellular uptake of these structures in HeLa cells, and the internalization of their cargo. The structures show minimal non-specific interaction with human serum albumin (HSA), a major protein component of blood. Moreover, BKM120-loaded DNA particles promote apoptosis in primary patient CLL lymphocytes and induce cell death when co-administered with doxorubicin in HeLa cells, without eliciting inflammation. Evaluation of this drug delivery system *in vivo* shows long circulation times up to 24 hours, full body distribution, high accumulation at tumor sites and minimal leakage through the blood–brain barrier. Our results demonstrate the great potential of these delivery vehicles as a general platform for chemotherapeutic drug delivery. We have previously shown that the DNA component of these structures is able to silence gene expression to a greater extent than DNA antisense structures alone, highlighting the promise of these DNA nanoparticles as combination small molecule and oligonucleotide therapeutics.^38^

## Results and discussion

### Synthesis of DNA nanoparticles

**Scheme 1:**
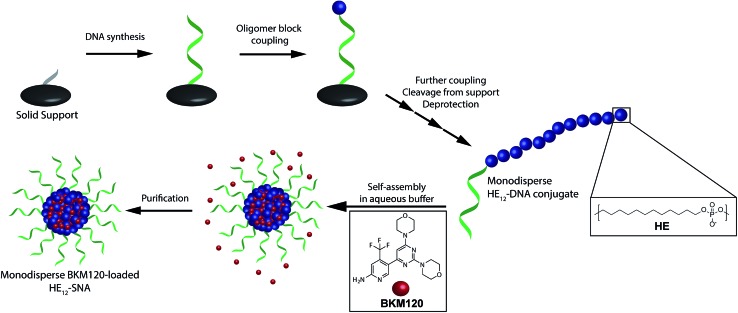
Schematic representation of the synthesis of DNA–polymer conjugates and BKM120 encapsulation method. Phosphoramidite monomers are attached to the 5′ end of the controlled glass pore (CPG) in a step-wise and sequence-controlled fashion. The 19-mer DNA strand is first built from the support, followed by 12 dodecane monomer additions (HE_12_) yielding monodisperse **HE_12_–DNA** conjugates. Self-assembly of the polymer–DNA conjugates in the presence of BKM120 and subsequent purification results in nearly monodisperse BKM120-loaded **HE_12_–SNAs**.

In order to construct a scalable and highly monodisperse drug delivery system, we generated a single type of DNA–polymer conjugates that self-assemble in aqueous buffer to form micellar DNA particles. These conjugates consist of a 19-mer DNA sequence attached to 12 dodecane (hexaethylene, HE) units (**HE_12_–DNA** conjugate, Scheme 1). HE_12_ units were appended to DNA by automated solid-phase synthesis using phosphoramidite chemistry.^27^ This approach offers monodisperse DNA–polymer conjugates in high yields and provides control over the length and sequence of the monomer units in the final structure. In our previous work, we showed that **HE_12_–DNA** conjugates self-assemble into highly monodisperse spherical nucleic acid particles (**HE_12_–SNAs**) in aqueous media containing divalent cations. These structures consist of an exterior DNA corona, and a hydrophobic HE_12_ core which provides a favourable environment for the entrapment of hydrophobic guest molecules. We also showed the encapsulation of a dye molecule, Nile Red, in the hydrophobic core of DNA nanoparticles.^27^ In this current study, we sought to test the encapsulation of a small-molecule protein kinase inhibitor, BKM120. We were interested in BKM120 because (1) despite its high potency, it suffers from deleterious side-effects in the CNS of patients. (2) The drug dimensions are compatible with the core size of the DNA nanoparticle system and (3) BKM120 exhibits an aqueous solubility of <1 mg ml^–1^, which makes it a suitable guest for our system.

### Evaluation of **HE_12_–SNAs** as BKM120 delivery vehicles

**Fig. 1:**
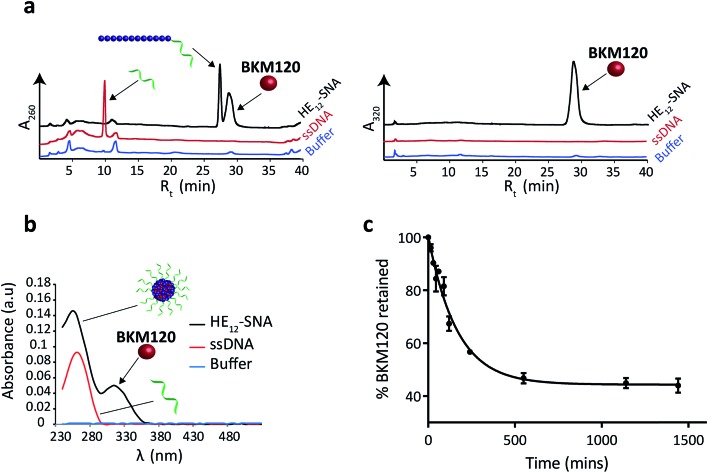
Evaluation of BKM120 encapsulation in **HE_12_–SNAs**. (a) Reversed phase HPLC analysis of **HE_12_–SNAs** (black), ssDNA control (red) and buffer control (blue) following drug purification. Detection at absorbance wavelength 260 nm (left panel) and 320 nm (right panel). The presence of a BKM120 peak solely in **HE_12_–SNA** samples suggests drug encapsulation. (b) UV-Vis measurements of BKM120-incubated **HE_12_–SNAs** (black), ssDNA control (red) and buffer control (blue) following purification. Drug encapsulation and loading capacity were determined by RP-HPLC and separately confirmed by UV-Vis measurements. The presence of a diagnostic drug peak at 320 nm in the **HE_12_–DNA** nanoparticle sample indicates drug encapsulation. (c) *In vitro* release of BKM120 loaded into **HE_12_–SNAs** studied by a dialysis method over 1 day at room temperature in 1× TAMg, measured in triplicate. Error bars represent the standard deviation of measurements.

To prepare BKM120-loaded **HE_12_–SNAs**, a solution of BKM120 in ethanol was allowed to evaporate forming a thin-film, which was then re-suspended into a solution of **HE_12_–DNA** conjugates in water, followed by the addition of assembly buffer and overnight thermal annealing (95–4 °C over 4 h). Thermal annealing was shown to yield less size-variability compared to overnight shaking at room temperature. Following the encapsulation process, the products were purified by size-exclusion chromatography and analyzed by reversed-phase HPLC (RP-HPLC) (Fig. 1a). The success of the purification method was critical to ensure complete removal of free-drug in solution. This was important for accurate determination of the nanoparticle drug-loading capacity and for further biological studies.

Data from RP-HPLC confirmed the encapsulation of BKM120 in **HE_12_–SNAs** in comparison to ssDNA and buffer controls. Traces were obtained at two different channels: a channel selective for DNA at 260 nm, and a BKM120-optimal channel at 320 nm. The co-elution of the DNA and BKM120 was only observed in **HE_12_–SNA** solutions, indicating the association of the drug with the structure. In the case of ssDNA, only a DNA peak was observed at 260 nm, reflecting the efficiency of the purification method at removing free drug in solution. The drug loading capacity of DNA nanoparticles was calculated from RP-HPLC data and separately confirmed by UV-Vis spectroscopy (Fig. 1b, see Fig. SF5†). For **HE_12_–SNAs**, the loading capacity was approximately 29% w/w, where ∼9 molecules of BKM120 were encapsulated per DNA–polymer conjugate strand. The aqueous solubility of BKM120 in the **HE_12_–SNAs** was enhanced to 24.4 μg ml^–1^, compared to <1 μg ml^–1^ in water. RP-HPLC was also used to calculate the recovered yield following purification. In general, ∼65% of the amount of DNA–polymer conjugates was retained following purification (see Fig. SF5†). Additionally, the drug-loaded structures had a shelf-life of over 4 weeks when stored at both room temperature and 4 °C (see Fig. SF7 and SF8†).

Having confirmed BKM120 encapsulation, we then studied the *in vitro* release kinetics of BKM120 in **HE_12_–SNAs** (Fig. 1c and SF6†). BKM120 release was evaluated by monitoring the decrease in concentration of the drug from a solution of loaded structures dialyzed against 1× TAMg at room temperature over 24 h. It was found that **HE_12_–SNAs** release BKM120 at a slow and sustained rate with ∼40% of the drug retained after 24 hours (Fig. 1c). The critical micellar concentration (CMC), above which **HE_12_–DNA** conjugates aggregate into **HE_12_–SNAs** was also studied. It was found that **HE_12_–DNA** conjugates aggregate with an associated CMC of 0.5 μM ± 0.2 μM in the presence of 12.5 mM Mg^2+^ (see Fig. SF9†).

**Fig. 2:**
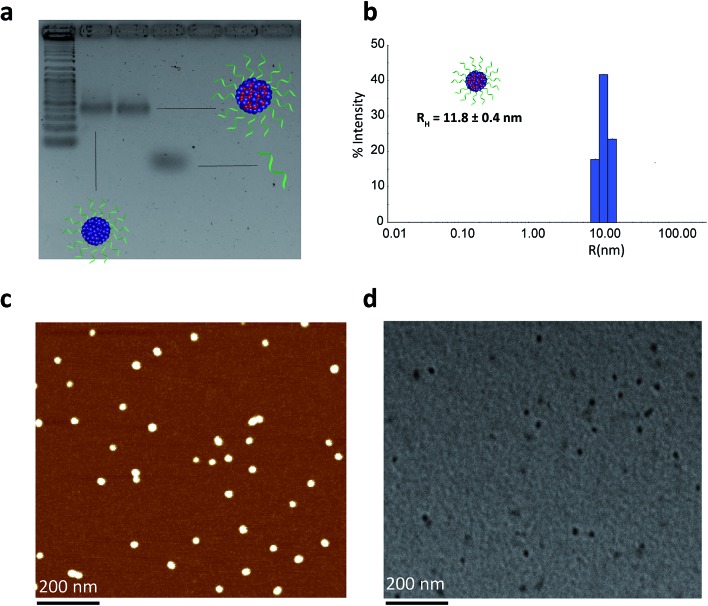
Structural characterization of BKM120-loaded **HE_12_–SNAs**. (a) Agarose gel electrophoresis (AGE) analysis of drug-loaded nanoparticles showing intact structures with no observed side products. (b) Dynamic light scattering (DLS) data showing a highly monodisperse population of drug-loaded nanoparticles in solution. (c) Atomic force microscopy (AFM) image showing a spherical nearly monodisperse population of drug-loaded products on surface. (d) Transmission electron microscopy (TEM) image of drug-loaded products reflecting highly monodisperse structures. Scale bars = 200 nm.

We then proceeded to characterize the BKM120-loaded products. The sizes of the nanoparticles were studied by agarose gel electrophoresis (AGE), dynamic light scattering (DLS), atomic force microscopy (AFM) and transmission electron microscopy (TEM) (Fig. 2). Data from AGE revealed the maintained structural integrity of BKM120-loaded particles with no observed side products (Fig. 2a). DLS analysis revealed a highly monodisperse population of drug-loaded structures in solution with a hydrodynamic radius of 11.8 ± 0.4 nm (Fig. 2b). AFM and TEM images demonstrated that BKM-120 loaded particles were well-dispersed on surface, with calculated dry-state diameter of 28 ± 4 nm and 21 ± 3 nm, respectively (Fig. 2c and d, SF11 & SF12†). The structures also appeared to retain high level of monodispersity, despite slightly widened features. The obtained dimensions are in agreement with solution measurements by DLS. The slightly widened morphology could be explained by the deposition of these structures on the surface and drying effects. The drug-loaded nanoparticles seemed to lose their spherical shape upon deposition confirmed by the lower height (8 nm) and slightly widened diameter as calculated by AFM.

### Stability of **HE_12_–SNAs**

**Fig. 3:**
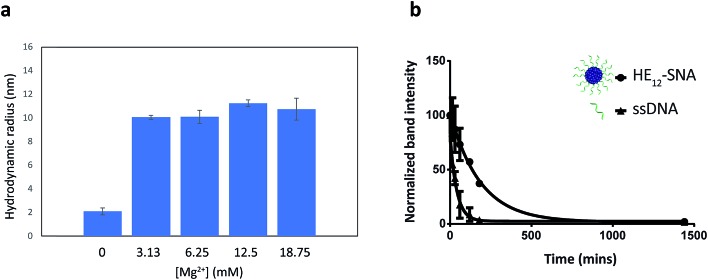
Stability of **HE_12_–SNAs** under biologically relevant conditions. (a) DLS histograms displaying the hydrodynamic radius of DNA nanoparticles under variations in magnesium concentrations in Tris buffer. DLS analysis show the maintained structural integrity of **HE_12_–DNA** particles under large variations of ionic conditions. Disassembly of the structure was only observed upon full ionic depletion. (b) Serum stability of the **HE_12_–SNAs** in biological conditions. **HE_12_–SNAs** have a measured half-life of 2.2 h which is 4.6 times higher than that of ssDNA. Error bars represent the standard deviation of measurements.

DNA nanostructures such as DNA origami and 3-dimensional nanoarchitectures purely composed of DNA, are typically assembled in buffers containing moderate concentrations of divalent metal cations (∼5–20 mM) in order to mask the electrostatic repulsion between DNA strands.^65–67^ Deviations from this window of buffer conditions can have devastating effects on the structures, causing shape distortion, aggregation or total collapse of structure. This limits the use of DNA nanostructures for biological applications. In our case, the assembly of **HE_12_–SNAs** was also shown to be dependent on the presence of divalent metal cations, however, the main driving force of assembly is hydrophobic interactions rather than Watson–Crick base-pairing. With this in mind, we sought to test whether our system can withstand variations in ionic concentrations and preserve structural identity in physiologically relevant environments. Evaluation of the nanoparticle stability in different buffer conditions was carried out by DLS measurements (Fig. 3). We tested concentration variations of two groups of candidates; divalent metal ions (Mg^2+^ and Ca^2+^) in Tris buffer (Fig. 3a and SF13–15†) and different titrations of Dulbecco's Phosphate Saline Buffer (DPBS with Mg^2+^ and Ca^2+^), a buffer used in cell culture (see Fig. SF16†). Data from DLS showed that **HE_12_–SNAs** could withstand large variations in ionic concentrations. At high ionic concentrations (18.75 mM Mg^2+^ and 2× DPBS), no structural aggregation was observed. Additionally, at Mg^2+^ concentrations as low as 0.25 mM (in 0.5× DPBS), the structures maintained their natural morphology with no observed disassembly. The structures were also compatible with a calcium-containing Tris buffer at concentrations similar to physiological plasma concentrations (∼1.2–1.5 mM).^68^ Only upon total depletion of divalent cations did the structures disassemble into monomeric **HE_12_–DNA** units (see Fig. SF17†). The enhanced stability in different buffer conditions can be partly attributed to hydrophobic interactions providing an additional cohesive force to preserve structural morphology.

We then proceeded to test the nuclease stability of our structures in 10% fetal bovine serum (FBS) solution at 37 °C (Fig. 3b, see Fig. SF18†). This was important because rapid nuclease degradation is a major challenge for DNA nanostructures as they are translated to the *in vitro* culture environment.^66^ We measured a half-life of 2.2 h for **HE_12_–SNAs**, which was 4.6-fold higher than the results obtained for ssDNA (28 min). This demonstrated the enhanced stability of our system against nuclease degradation and could be due to the dense packing of DNA creating a steric barrier. We further assessed the serum stability in phosphorothioated DNA nanoparticles, where the nonbridging oxygen in the phosphate backbone of the DNA strand was replaced with a sulfur. This simple modification resulted in nuclease resistance for over 72 hours (Fig. SF19†), and could be easily implemented in our system as facile strategy for enhanced stability.

### Cellular uptake of non-transfected **HE_12_–SNAs**

**Fig. 4:**
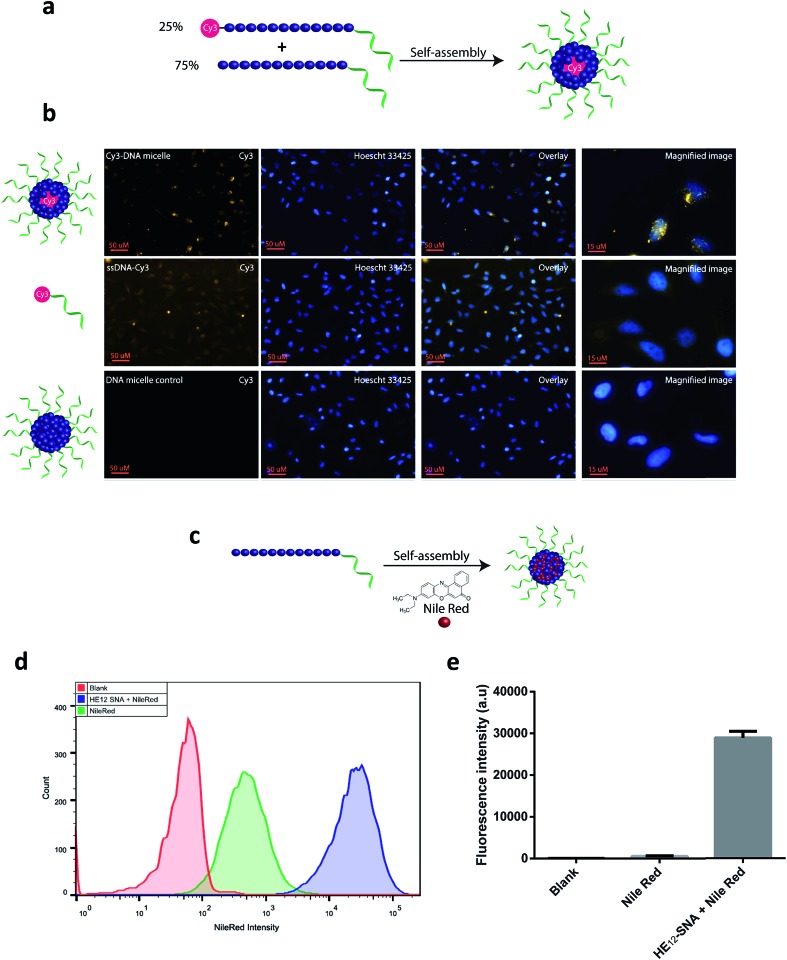
Cellular localization of **HE_12_–DNA** nanoparticles and encapsulated cargo. (a) Preparation of Cy3–labeled nanoparticles. Cy3–**HE_12_–DNA** and **HE_12_–DNA** were mixed in 25 : 75 molar ratios to generate nearly monodisperse Cy3-labeled **HE_12_–DNA** nanoparticles. (b) Confocal microscopy images demonstrating the cellular uptake of Cy3-labeled particles in HeLa cells after 24 hour incubation. (c) Preparation of Nile Red-loaded DNA nanoparticles. (d) Flow cytometry measurements showing the increased uptake of Nile Red when delivered by **HE_12_–SNAs**. All samples were incubated for 12 hours, [Nile Red] = 375 nM in cell culture media. Nile Red images were acquired using exc. 516 nm and YellowG_670/30 filter. (e) Quantification of Nile Red intensity measured by flow cytometry. All measurements were performed in triplicates, and the error bars represent the standard deviation of measurements.

The *in vitro* cellular uptake and internalization of **HE_12_–SNAs** were studied by confocal fluorescence microscopy. As a first step, we generated Cy3-labeled DNA nanoparticles (see Fig. SF20†). This was achieved by mixing Cy3–**HE_12_–DNA** (where Cy3 was attached to the HE_12_ polymer at the opposite end of the DNA) and unlabeled **HE_12_–DNA** conjugates in 25 : 75 molar ratios, followed by thermal annealing, 95–4 °C over 4 hours (Fig. 4a). This approach yielded highly monodisperse dye-labeled nanoparticles with the dye molecules embedded in the core. This was important as surface projection of a lipophilic dye molecule could alter the uptake profile of the nanoparticles through cell membranes. Following 24 hour incubation in HeLa cells, fluorescence data indicated the high cellular uptake of Cy3–DNA nanoparticles and localization in the cytoplasm in the perinuclear region (Fig. 4b). Several intense foci were observed indicating the high efficiency of uptake.

We were then interested in studying the internalization of encapsulated cargo. Knowing that BKM120 has poor fluorescence properties, we decided to monitor the uptake of a fluorescent dye, Nile Red, encapsulated in our DNA nanostructures (Fig. 4c, see Fig. SF21†). The encapsulation of Nile Red further demonstrates the versatility of this delivery system for accommodating different guest molecules, highlighting its potential application as a general drug delivery platform. HeLa (adenocarcinoma) cells were incubated with Nile Red loaded-nanoparticles, Nile Red alone or DNA nanoparticle control at 37 °C. Flow cytometry was used to quantify the amount of Nile Red uptake by HeLa cells (Fig. 4d). After 12 hours of incubation and several washing steps, analysis of the flow cytometry data revealed significantly higher intracellular fluorescence of Nile Red when delivered by **HE_12_–DNA** nanoparticles compared to low non-specific internalization of Nile Red control (Fig. 4d, e and SF22†). The higher uptake of Nile Red was also confirmed by confocal fluorescence microscopy, where the dye was mostly observed in the cytoplasm in the perinuclear region, confirming high uptake efficiency (see Fig. SF23†). Taken together, these experiments suggest that the increase in Nile Red uptake is due to its encapsulation and internalization by **HE_12_–DNA** nanoparticles.

### Cell toxicity of BKM120-loaded **HE_12_–SNAs**

**Fig. 5:**
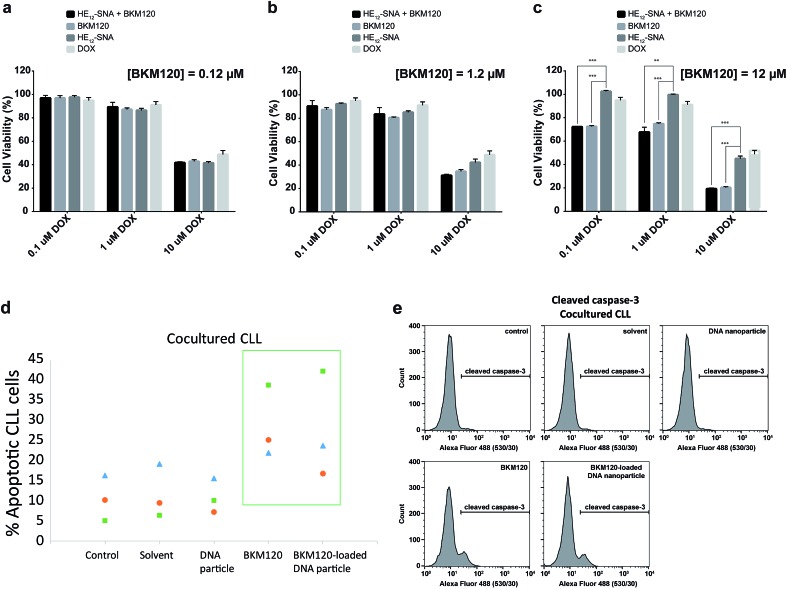
*In vitro* cellular toxicity of BKM120-loaded **HE_12_–SNAs**. Cytotoxicity of BKM120-loaded nanoparticles when administered at concentrations (a) 0.12 μM (b) 1.2 μM (c) 12 μM prior to doxorubicin treatment, measured over 24 hours. *** corresponds to *p* < 0.0001 and ** to *p* < 0.001. Error bars represent the standard deviation of measurements. (d) Annexin/PI staining and (e) cleaved caspase-3 assay showing the potency of BKM120-loaded particles at inducing apoptosis in primary patient B-CLL lymphocytes in the presence of the BMS2 stromal cells support (cocultured CLL), analyzed by flow cytometry.

Based on the cellular uptake and dye internalization studies in HeLa cells, we were interested if the higher uptake of our nanostructures would correlate to increased therapeutic activity of the drug-loaded constructs. The *in vitro* cytotoxicity of BKM120-loaded **HE_12_–SNAs** was measured against human cervical cancer (HeLa) cells. Cytotoxicity was evaluated by comparing a dose-dependent administration of BKM120 in DNA nanoparticles with naked BKM120 and DNA particles as controls (see Fig. SF24†). In the tested concentration range, **HE_12_–DNA** particles loaded with BKM120 showed low cellular death in HeLa cells. Based on these results, we then tested the synergistic effect of loaded BKM120 in combination with doxorubicin (Dox). Our platform acting as a sensitizer in HeLa cells also highlights the versatility of our system as a general drug delivery system for anticancer drugs. For this study, HeLa cells were initially sensitized with three different concentrations of BKM120-loaded particles prior to incubation with various Dox dosages (Fig. 5a–c). Interestingly, as illustrated in Fig. 5, the cytotoxicity of Dox was enhanced upon co-administration with BKM120-loaded particles in a dose-dependent manner. The effect was most pronounced at higher Dox concentrations (Fig. 5c). The reduced cytotoxic differences between loaded BKM120 *versus* its un-encapsulated form, is in part due to the lipophilic nature of the drug which can diffuse passively through cell membranes and cause cytotoxicity. However, we anticipate that this nanocarrier platform could provide advantages in the delivery of BKM120 and other chemotherapeutic drugs by altering their *in vivo* delivery profile. Additionally, the capability of functionalizing **HE_12_–SNAs** with targeting ligands could also limit some of the drug's manifested side-effects and provide a targeted delivery regimen in tumors.

*In vitro* studies on BKM120 have shown this drug to induce cell death in B-CLL cells and promote apoptosis.^63^ Thus, we asked whether BKM120-loaded particles can promote cell apoptosis. To address this question, we investigated the induction of apoptosis through Annexin V/propidium iodide (PI) staining assay in primary patient Chronic Lymphocytic Leukemia (CLL) lymphocytes (Fig. 5d). Earlier studies have shown that stromal cells induce drug resistance and promote cell survival through secretion of chemokines and cell–cell interaction.^69^ Additionally, the bone marrow microenvironment has shown to prevent apoptosis in primary CLL lymphocytes by modulating the PI3K/Akt pathway.^70^ As expected, the stromal microenvironment model (BSM2 stromal cells) protected CLL lymphocytes from spontaneous apoptosis as seen with the untreated controls (Fig. 5d). We found that BKM120-loaded structures promoted apoptosis in primary BMS2 cocultured CLL lymphocytes from 3 different patients, 24 hours after treatment. To further confirm these findings, we monitored the cleavage of caspase-3, a catalytic step in the apoptotic pathway. In accordance with the Annexin V/PI analysis, BKM120-loaded particles induced caspase-3 activity in CLL lymphocytes; both in the presence and absence of BMS2, confirming their enhanced activity in complex patient cellular environments (Fig. 5e and SF25†).

**Fig. 6:**
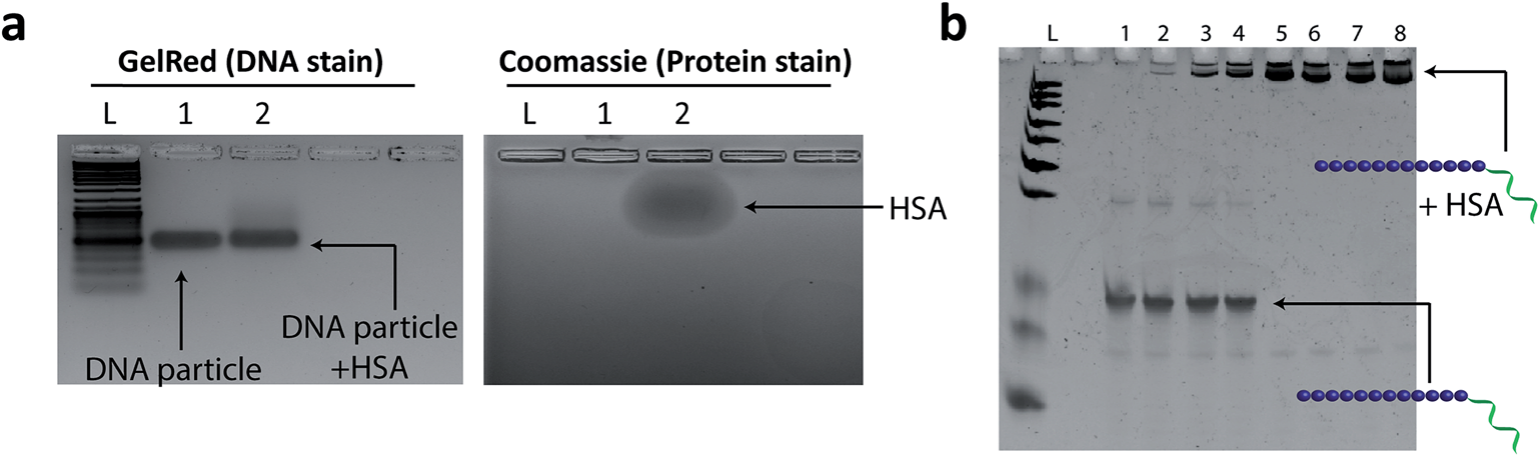
Evaluating the interaction of **HE_12_–SNAs** with human serum albumin (HSA). (a) Agarose gel electrophoresis of **HE_12_–SNAs** prior (Lane 1) and post incubation (Lane 2) with HSA. The gels were visualized under Gel Red DNA stain channel (left panel), Coomassie Blue protein stain (right panel). GelRed panel shows the absence of a gel mobility shift of DNA nanoparticles after HSA incubation. Coomassie panel displays the lower mobility shift of HSA protein compared to DNA nanoparticles. (b) Denaturing PAGE analysis of dissembled **HE_12_–DNA** conjugate strands titrated with different HSA concentrations. Lane 1: **HE_12_–DNA** strand control, Lanes 2–8, HSA dilutions of 1/1000, 1/100, 1/50, 1/10, 1/5, 1/2 and undiluted HSA (526 μM stock). Under denaturing conditions, disassembly of the DNA particle exposes the lipophilic HE_12_ segments, which in turn results in strong binding to HSA protein.

To evaluate the potential immunogenicity of this delivery system, we investigated the effect of **HE_12_–SNAs** on TNF-alpha induction (see Fig. SF26†). TNF-alpha is a signalling protein involved in systemic inflammation.^71^ Higher levels of this protein indicate an elicited immune response. We tested our system in comparison to lipopolysaccharide (LPS) and a synthetic dsRNA (PolyIC) which have been reported to induce the expression TNF-alpha.^72,73^ It has also been reported that the time course of TNF-alpha induction shows a rise-and decline profile with peak elevation at 2–6 hours post exposure.^74,75^ As illustrated in Fig. 6, after 5 hours of incubation **HE_12_–SNAs** exhibited no systemic inflammation with very low levels of TNF-alpha induction compared to LPS and PolyIC. The effect becomes less pronounced at the 12 and 24 hour mark. This result supports the non-immunogenic nature of **HE_12_–SNAs**.

### Nanoparticle interaction with serum proteins

Previous reports have demonstrated that inert polymers such as hydrophilic polyethylene glycol chains improve the efficacy of encapsulated drugs by reducing *in vivo* opsonisation with serum proteins, mainly human serum albumin (HSA).^76^ This not only prevents the rapid recognition of structures by the reticuloendothelial system (RES), but also provides prolonged blood circulation of nanostructures and higher accumulation at targeted sites.^77–79^ In our case, we hypothesized that the dense hydrophilic DNA outer shell would provide a surface unfavorable for binding HSA protein. **HE_12_–SNAs** were pre-assembled then incubated with a 5× molar excess of HSA for 2 hours at room temperature, and analyzed by agarose gel electrophoresis (AGE) (Fig. 6). Since HSA exhibits lower mobility on gel compared to DNA nanoparticles (Fig. 6a), an association with the protein should results in a gel mobility shift of the structures. As illustrated in Fig. 6a, following incubation, no interaction was observed between the DNA particles and HSA protein (GelRed channel). It appears that the outer DNA shell dictates the interaction between HSA and the DNA structures. In a control experiment, the micellar DNA structures were denatured by the addition of a solution of urea and depletion of magnesium cations prior to HSA addition, exposing their long aliphatic chains (Fig. 6b and SF27†). In this case, even at low protein concentrations, HSA was observed to strongly bind to the DNA–polymer conjugates. These findings suggest that the outer hydrophilic ssDNA corona limits albumin adsorption, and indicates that the DNA structures remain stable upon exposure to the protein. In addition, we used Nile Red-loaded particles as a visual tool along with gel electrophoresis to further confirm the lack of interaction between DNA nanoparticles and HSA (Fig. SF28 and SF29†).

### *In vivo* biodistribution of **HE_12_–SNAs**

**Fig. 7:**
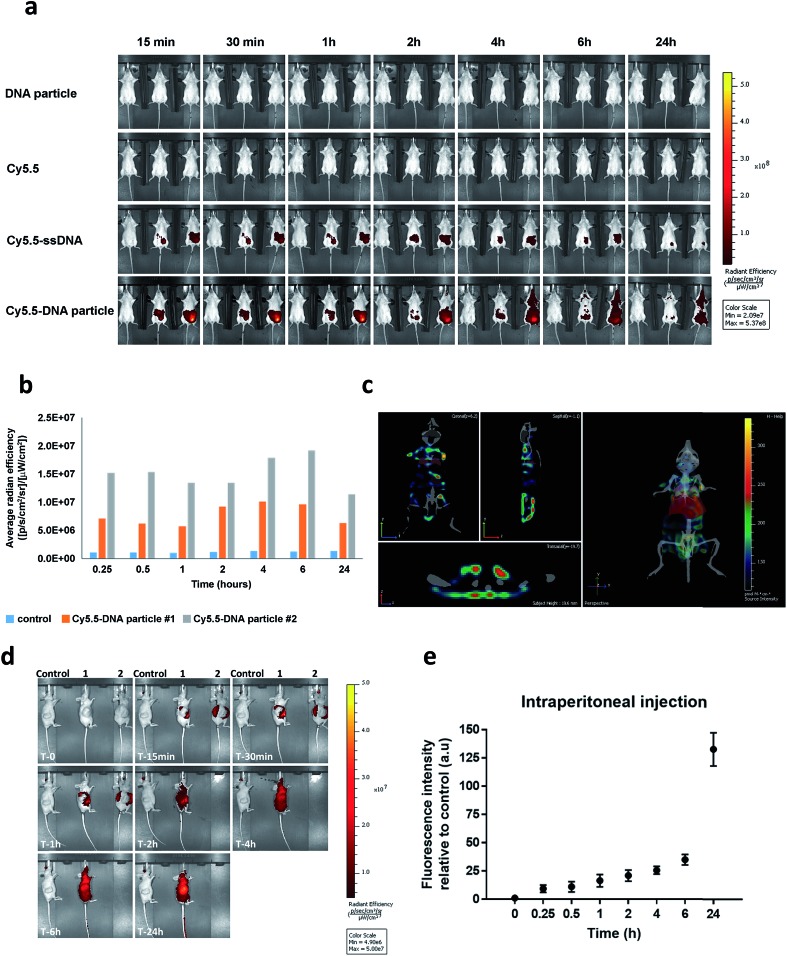
*In vivo* biodistribution of Cy5.5-labeled **HE_12_–DNA** nanoparticles. (a) Cy5.5 fluorescence data overlaid on X-ray images measured over time after intraperitoneal injection. Top: unlabeled **HE_12_–DNA** nanoparticle, 2^nd^ top: Cy5.5 dye molecule, 2^nd^ bottom: Cy5.5–ssDNA, bottom: Cy5.5–**HE_12_–DNA** nanoparticle. (b) Quantified biodistribution data of Cy5.5 intensity measured as a function of time for Cy5.5–**HE_12_–DNA** nanoparticles. (c) 3D full body fluorescence scan at 24 h, overlaid with body organs (liver highlighted in red). (d) Biodistribution of Cy5.5-labeled DNA nanoparticles in HCT116 colon cancer xenograft. Control: treated with unlabeled particle, 1 & 2: treated with Cy5.5–DNA particles. Cy5.5 fluorescence data overlaid on X-ray images measured over time following subcutaneous injection. (e) Quantified fluorescence intensity of Cy5.5–**HE_12_–DNA** particles at the tumor site measured over time. Error bars represent the standard deviation of measurements.

To our knowledge, the *in vivo* behaviour of polymeric DNA nanoparticles has not been previously studied. With that in mind, we proceeded with an *in vivo* screening *via* optical imaging which would allow for real-time tracking and overall biodistribution profiles. For this purpose, highly monodisperse Cy5.5–**HE_12_–DNA** nanoparticles were prepared which contained the dye molecule in their core. This was achieved by mixing Cy5.5–**HE_12_–DNA** (where Cy5.5 was attached to the HE_12_ polymer at the opposite end of the DNA) and unlabeled **HE_12_–DNA** conjugates in 25 : 75 molar ratios, followed by thermal annealing (95–4 °C over 4 hours) (see Fig. SF30 and SF31†). Nanoparticle biodistribution was evaluated by fluorescence imaging, following intraperitoneal injection (Fig. 7a and b) and intravenous administration of Cy5.5-labeled structures measured over 24 hours (see Fig. SF32†). Remarkably, Cy5.5-labeled DNA particles showed full-body distribution with long circulation times up to 24 hours (Fig. 7a–c and SF32†). Control experiments using Cy5.5-labeled single stranded DNA, showed loss of fluorescence, most likely due to DNA degradation. Similarly, the dye only Cy5.5 sample showed immediate loss of fluorescence, likely because of its insolubility. In contrast, the prolonged fluorescence biodistribution of Cy5.5–**HE_12_–DNA** nanoparticles could indicate very slow structural degradation in the blood stream. This behavior was further observed with intravenous injection of Cy5.5–**HE_12_–SNAs** (see Fig. SF32†). Compared to Cy5.5–ssDNA which showed rapid decrease in signal after 30 minutes, Cy5.5–**HE_12_–SNA** exhibited a delayed decrease starting at 6 hours. These results also corroborate our *in vitro* experiments that demonstrate enhanced stability of these DNA structures under physiological conditions (Fig. 3 and 4), and could also indicate that the DNA portion of Cy5.5–**HE_12_–SNA** is more shielded as the nanostructure circulates in the body. Interestingly, at the 6 hour mark, the rate of signal decrease in both Cy5.5–ssDNA and Cy5.5–**HE_12_–SNAs** appear to be similar which could indicate that at this point, the DNA portion of Cy5.5–**HE_12_–SNAs** may be degraded, and the remaining Cy5.5–HE_12_ portion behaves similarly to the remaining Cy5.5 dye in the Cy5.5–ssDNA sample.

Further 3D fluorescence imaging, which highlights organ-specific distribution, indicated low levels of excretion (liver and kidney) (Fig. 7c). Notably, low levels of fluorescence were also observed in brain and lungs after 2 and 24 hours (see ESI video clips 1 & 2†). The biodistribution within the blood stream without accumulation in non-specific organs, particularly in the brain where BKM120 manifests side-effects, is important to decrease adverse effects observed during systemic drug treatments.

The next question was to test whether our DNA nanostructures could reach and accumulate in solid tumors. To address that, the biodistribution of Cy5.5-labeled structures was evaluated in a cancer xenograft model. In our hands, the success of forming CLL xenografts was hampered by the inefficient engraftment of MEC-1 (CLL cell line) into rag2–/–γc–/–mice. Compared to CLL, colon cancer xenografts formed solid tumors much more efficiently in our mice model. Therefore, HCT116 colon cancer xenografts were used as a model system to test the biodistribution of our DNA nanostructures. Previous reports have demonstrated that nanoparticles tend to accumulate in higher levels in tumor tissue, a phenomenon known as the enhanced permeation and retention (EPR) effect.^80,81^ In our case, we measured the accumulation of nanoparticles at the tumor site over time following intraperitoneal injection (Fig. 7d). Cy5.5-labeled DNA particles showed a steady increase in accumulation at tumor sites for up to 24 hours (Fig. 7e). High accumulation in tumors was also observed under intravenous administration. As expected, Cy5.5-labeled structures exhibited higher diffusion rates compared to intraperitoneal delivery with accumulation peaking at 6 h (Fig. SF33†). The steady increase in accumulation of Cy5.5-labeled particles at the tumor site is predicted to translate into the same pattern of anticancer drug delivery by **HE_12_–DNA** structures, which will provide an important mechanism to minimize potential complications of this drug. Overall, the *in vivo* stability and biodistribution profiles of **HE_12_–DNA** nanoparticles highlight their great potential as a robust drug delivery system.

## Conclusion

We have developed a highly monodisperse DNA nanoparticle delivery platform for small-molecule chemotherapeutics. Our structures show effective loading and slow release of BKM120, and have a long shelf-life. The DNA nanoparticles are made of monodisperse, sequence-defined polymers units, they are stable under physiological ionic concentrations, and exhibit increased resistance to nucleases in biological environments. Furthermore, these structures demonstrate efficient uptake in cancer cells, and increased internalization of cargo. *In vitro* studies show the ability of BKM120-loaded particles to induce cellular death and apoptosis, including synergistic effects between BKM120 and antitumor drugs, without causing non-specific inflammation. Moreover, the absence of interaction with HSA can possibly protect the structures from RES uptake. Further investigation of the *in vivo* biodistribution of DNA nanoparticles demonstrates full-body distribution and long circulation times of these structures. Furthermore, the particles are not observed to cross the blood–brain barrier an important feature towards limiting the side-effects of BKM120 or any drug molecule with CNS off-target activity. The structures also show high tumor accumulation in xenograft models highlighting their potential for targeted cancer therapy.

Given our findings, **HE_12_–DNA** nanoparticles show great promise as delivery vehicles for chemotherapeutics. This initial work has demonstrated the ability to load drugs and protect them in different biological conditions, achieve *in vitro* activity in primary patient cell lines, and monitor the *in vivo* biodistribution of these structures in mice to understand their real-time trafficking and stability. Future studies on this platform will focus on adapting cross-linking strategies to enhance drug loading capacity, retention and structural stability *in vivo*. Additionally, taking advantage of the DNA shell, surface modifications such as targeting ligands and oligonucleotide therapeutics will be implemented. We envisage this system to see applications in targeted cancer therapy and delivery of combinational small-molecule and oligonucleotide therapeutics.

## References

[cit1] Hallek M. (2009). Hematology Am. Soc. Hematol. Educ. Program.

[cit2] Peer D., Karp J. M., Hong S., Farokhzad O. C., Margalit R., Langer R. (2007). Nat. Nanotechnol..

[cit3] Kukowska-Latallo J. F., Candido K. A., Cao Z., Nigavekar S. S., Majoros I. J., Thomas T. P., Balogh L. P., Khan M. K., Baker J. R. (2005). Cancer Res..

[cit4] Symon Z., Peyser A., Tzemach D., Lyass O., Sucher E., Shezen E., Gabizon A. (1999). Cancer.

[cit5] Danson S., Ferry D., Alakhov V., Margison J., Kerr D., Jowle D., Brampton M., Halbert G., Ranson M. (2004). Br. J. Cancer.

[cit6] Nakanishi T., Fukushima S., Okamoto K., Suzuki M., Matsumura Y., Yokoyama M., Okano T., Sakurai Y., Kataoka K. (2001). J. Controlled Release.

[cit7] Flenniken M. L., Liepold L. O., Crowley B. E., Willits D. A., Young M. J., Douglas T. (2005). Chem. Commun..

[cit8] Ren Y., Wong S. M., Lim L. Y. (2007). Bioconjugate Chem..

[cit9] Hirsch L. R., Stafford R. J., Bankson J. A., Sershen S. R., Rivera B., Price R. E., Hazle J. D., Halas N. J., West J. L. (2003). Proc. Natl. Acad. Sci. U. S. A..

[cit10] Kam N. W., O'Connell M., Wisdom J. A., Dai H. (2005). Proc. Natl. Acad. Sci. U. S. A..

[cit11] Ediriwickrema A., Saltzman W. M. (2015). ACS Biomater. Sci. Eng..

[cit12] Petros R. A., DeSimone J. M. (2010). Nat. Rev. Drug Discovery.

[cit13] Choi H. S., Liu W., Liu F., Nasr K., Misra P., Bawendi M. G., Frangioni J. V. (2010). Nat. Nanotechnol..

[cit14] De Jong W. H., Borm P. J. A. (2008). Int. J. Nanomed..

[cit15] Mu Q., Jiang G., Chen L., Zhou H., Fourches D., Tropsha A., Yan B. (2014). Chem. Rev..

[cit16] Prabhu R. H., Patravale V. B., Joshi M. D. (2015). Int. J. Nanomed..

[cit17] Kwon G. S., Naito M., Kataoka K., Yokoyama M., Sakurai Y., Okano T. (1994). Colloids Surf., B.

[cit18] Savic R., Eisenberg A., Maysinger D. (2006). J. Drug Targeting.

[cit19] Kwak M., Herrmann A. (2011). Chem. Soc. Rev..

[cit20] Vyborna Y., Vybornyi M., Rudnev A. V., Häner R. (2015). Angew. Chem., Int. Ed..

[cit21] Zhao Z., Wang L., Liu Y., Yang Z., He Y.-M., Li Z., Fan Q.-H., Liu D. (2012). Chem. Commun..

[cit22] Alemdaroglu F. E., Alemdaroglu N. C., Langguth P., Herrmann A. (2008). Adv. Mater..

[cit23] Mirkin C. A., Letsinger R. L., Mucic R. C., Storhoff J. J. (1996). Nature.

[cit24] Rosi N. L., Giljohann D. A., Thaxton C. S., Lytton-Jean A. K., Han M. S., Mirkin C. A. (2006). Science.

[cit25] Jensen S. A., Day E. S., Ko C. H., Hurley L. A., Luciano J. P., Kouri F. M., Merkel T. J., Luthi A. J., Patel P. C., Cutler J. I., Daniel W. L., Scott A. W., Rotz M. W., Meade T. J., Giljohann D. A., Mirkin C. A., Stegh A. H. (2013). Sci. Transl. Med..

[cit26] Wang X., Hao L., Bu H. F., Scott A. W., Tian K., Liu F., De Plaen I. G., Liu Y., Mirkin C. A., Tan X. D. (2016). Sci. Rep..

[cit27] Edwardson T. G., Carneiro K. M., Serpell C. J., Sleiman H. F. (2014). Angew. Chem., Int. Ed..

[cit28] Burns J. R., Göpfrich K., Wood J. W., Thacker V. V., Stulz E., Keyser U. F., Howorka S. (2013). Angew. Chem., Int. Ed..

[cit29] Krishnan S., Ziegler D., Arnaut V., Martin T. G., Kapsner K., Henneberg K., Bausch A. R., Dietz H., Simmel F. C. (2016). Nat. Commun..

[cit30] Fendt L.-A., Bouamaied I., Thöni S., Amiot N., Stulz E. (2007). J. Am. Chem. Soc..

[cit31] Schreiber R., Do J., Roller E.-M., Zhang T., Schuller V. J., Nickels P. C., Feldmann J., Liedl T. (2014). Nat. Nanotechnol..

[cit32] Zhang Q., Jiang Q., Li N., Dai L., Liu Q., Song L., Wang J., Li Y., Tian J., Ding B., Du Y. (2014). ACS Nano.

[cit33] Rothemund P. W. K. (2006). Nature.

[cit34] Chien M.-P., Rush A. M., Thompson M. P., Gianneschi N. C. (2010). Angew. Chem., Int. Ed..

[cit35] Zhang F., Zhang S., Pollack S. F., Li R., Gonzalez A. M., Fan J., Zou J., Leininger S. E., Pavía-Sanders A., Johnson R., Nelson L. D., Raymond J. E., Elsabahy M., Hughes D. M. P., Lenox M. W., Gustafson T. P., Wooley K. L. (2015). J. Am. Chem. Soc..

[cit36] Wu Y., Sefah K., Liu H., Wang R., Tan W. (2010). Proc. Natl. Acad. Sci. U. S. A..

[cit37] Kedracki D., Maroni P., Schlaad H., Vebert-Nardin C. (2014). Adv. Funct. Mater..

[cit38] Fakhoury J. J., Edwardson T. G., Conway J. W., Trinh T., Khan F., Barlog M., Bazzi H. S., Sleiman H. F. (2015). Nanoscale.

[cit39] Jeong J. H., Park T. G. (2001). Bioconjugate Chem..

[cit40] Rush A. M., Nelles D. A., Blum A. P., Barnhill S. A., Tatro E. T., Yeo G. W., Gianneschi N. C. (2014). J. Am. Chem. Soc..

[cit41] Li Z., Zhang Y., Fullhart P., Mirkin C. A. (2004). Nano Lett..

[cit42] Serpell C. J., Edwardson T. G. W., Chidchob P., Carneiro K. M. M., Sleiman H. F. (2014). J. Am. Chem. Soc..

[cit43] Chidchob P., Edwardson T. G. W., Serpell C. J., Sleiman H. F. (2016). J. Am. Chem. Soc..

[cit44] Trinh T., Chidchob P., Bazzi H. S., Sleiman H. F. (2016). Chem. Commun..

[cit45] Alemdaroglu F. E., Ding K., Berger R., Herrmann A. (2006). Angew. Chem., Int. Ed..

[cit46] Alemdaroglu F. E., Alemdaroglu N. C., Langguth P., Herrmann A. (2008). Macromol. Rapid Commun..

[cit47] Hallek M. (2015). Am. J. Hematol..

[cit48] Rai K. R., Peterson B. L., Appelbaum F. R., Kolitz J., Elias L., Shepherd L., Hines J., Threatte G. A., Larson R. A., Cheson B. D., Schiffer C. A. (2000). N. Engl. J. Med..

[cit49] Hillmen P., Skotnicki A. B., Robak T., Jaksic B., Dmoszynska A., Wu J., Sirard C., Mayer J. (2007). J. Clin. Oncol..

[cit50] O'Brien S. M., Kantarjian H. M., Cortes J., Beran M., Koller C. A., Giles F. J., Lerner S., Keating M. (2001). J. Clin. Oncol..

[cit51] Keating M. J., O'Brien S., Albitar M., Lerner S., Plunkett W., Giles F., Andreeff M., Cortes J., Faderl S., Thomas D., Koller C., Wierda W., Detry M. A., Lynn A., Kantarjian H. (2005). J. Clin. Oncol..

[cit52] Wierda W., O'Brien S., Wen S., Faderl S., Garcia-Manero G., Thomas D., Do K. A., Cortes J., Koller C., Beran M., Ferrajoli A., Giles F., Lerner S., Albitar M., Kantarjian H., Keating M. (2005). J. Clin. Oncol..

[cit53] Tam C. S., O'Brien S., Wierda W., Kantarjian H., Wen S., Do K. A., Thomas D. A., Cortes J., Lerner S., Keating M. J. (2008). Blood.

[cit54] Datta S. R., Brunet A., Greenberg M. E. (1999). Genes Dev..

[cit55] Alessi D. R., James S. R., Downes C. P., Holmes A. B., Gaffney P. R., Reese C. B., Cohen P. (1997). Curr. Biol..

[cit56] Cuni S., Perez-Aciego P., Perez-Chacon G., Vargas J. A., Sanchez A., Martin-Saavedra F. M., Ballester S., Garcia-Marco J., Jorda J., Durantez A. (2004). Leukemia.

[cit57] Stokoe D., Stephens L. R., Copeland T., Gaffney P. R., Reese C. B., Painter G. F., Holmes A. B., McCormick F., Hawkins P. T. (1997). Science.

[cit58] Datta S. R., Dudek H., Tao X., Masters S., Fu H., Gotoh Y., Greenberg M. E. (1997). Cell.

[cit59] Cardone M. H., Roy N., Stennicke H. R., Salvesen G. S., Franke T. F., Stanbridge E., Frisch S., Reed J. C. (1998). Science.

[cit60] Kops G. J. P. L., Burgering B. M. T. (2000). J. Anat..

[cit61] Maira S. M., Pecchi S., Huang A., Burger M., Knapp M., Sterker D., Schnell C., Guthy D., Nagel T., Wiesmann M., Brachmann S., Fritsch C., Dorsch M., Chene P., Shoemaker K., De Pover A., Menezes D., Martiny-Baron G., Fabbro D., Wilson C. J., Schlegel R., Hofmann F., Garcia-Echeverria C., Sellers W. R., Voliva C. F. (2012). Mol. Cancer Ther..

[cit62] Brachmann S. M., Kleylein-Sohn J., Gaulis S., Kauffmann A., Blommers M. J. J., Kazic-Legueux M., Laborde L., Hattenberger M., Stauffer F., Vaxelaire J., Romanet V., Henry C., Murakami M., Guthy D. A., Sterker D., Bergling S., Wilson C., Brümmendorf T., Fritsch C., Garcia-Echeverria C., Sellers W. R., Hofmann F., Maira S.-M. (2012). Mol. Cancer Ther..

[cit63] Amrein L., Shawi M., Grenier J., Aloyz R., Panasci L. (2013). Int. J. Cancer.

[cit64] Bendell J. C., Rodon J., Burris H. A., de Jonge M., Verweij J., Birle D., Demanse D., De Buck S. S., Ru Q. C., Peters M., Goldbrunner M., Baselga J. (2012). J. Clin. Oncol..

[cit65] Woo S., Rothemund P. W. (2014). Nat. Commun..

[cit66] Hahn J., Wickham S. F., Shih W. M., Perrault S. D. (2014). ACS Nano.

[cit67] Gerling T., Wagenbauer K. F., Neuner A. M., Dietz H. (2015). Science.

[cit68] Fogh-Andersen N., Christiansen T. F., Komarmy L., Siggaard-Andersen O. (1978). Clin. Chem..

[cit69] Hayden R. E., Pratt G., Roberts C., Drayson M. T., Bunce C. M. (2012). Leuk. Lymphoma.

[cit70] Shehata M., Schnabl S., Demirtas D., Hilgarth M., Hubmann R., Ponath E., Badrnya S., Lehner C., Hoelbl A., Duechler M., Gaiger A., Zielinski C., Schwarzmeier J. D., Jaeger U. (2010). Blood.

[cit71] Locksley R. M., Killeen N., Lenardo M. J. (2001). Cell.

[cit72] van der Bruggen T., Nijenhuis S., van Raaij E., Verhoef J., Sweder van Asbeck B. (1999). Infect. Immun..

[cit73] North R. J., Dunn P. L., Havell E. A. (1991). J. Interferon Res..

[cit74] Michishita M., Yoshida Y., Uchino H., Nagata K. (1990). J. Biol. Chem..

[cit75] Shemi D., Azab A. N., Kaplanski J. (2000). J. Endotoxin Res..

[cit76] Li S.-D., Huang L. (2010). J. Controlled Release.

[cit77] Allen T. M. (1994). Trends Pharmacol. Sci..

[cit78] Owens Iii D. E., Peppas N. A. (2006). Int. J. Pharm..

[cit79] Ishida T., Kirchmeier M. J., Moase E. H., Zalipsky S., Allen T. M. (2001). Biochim. Biophys. Acta.

[cit80] Matsumura Y., Maeda H. (1986). Cancer Res..

[cit81] Torchilin V. (2011). Adv. Drug Delivery Rev..

