# Supplementary material for: Precision spherical nucleic acids for delivery of anticancer drugs
Source: Chem Sci. 2017 Jul 5;8(9):6218–29. doi: 10.1039/c7sc01619k (PMC5628336; doi:10.1039/c7sc01619k)
Supplement: Supplementary file 3 [file SC-008-C7SC01619K-s003.pdf]

Electronic Supplementary Material (ESI) for Chemical Science.
This journal is © The Royal Society of Chemistry 2017

Precision Spherical Nucleic Acids for Delivery of Anticancer Drugs

Danny Bousmail,[a] Lilian Amrein,[b] Johans J. Fakhoury,[a] Hassan H. Fakih,[a] John C.C. Hsu,[a] Lawrence Panasci[b]* and Hanadi F. Sleiman[a]*

[a] Department of Chemistry and Centre for Self-Assembled Chemical Structures (CSACS), McGill University, 801 Sherbrooke St. W., Montreal, Canada

[b] Department of Oncology, Jewish General Hospital, 3755 Cote Sainte-Catherine Rd., Montreal, Canada

Supporting Information

Content

I.	General	S2
II.	Instrumentation	S3
III.	Solid-phases synthesis and purification	S4
IV.	Sequences of DNA-polymer conjugates and characterization	S5
V.	Evaluation of the encapsulation of BKM120	S9
VI.	Shelf-life of BKM120-loaded nanoparticles	S12
VII.	Determination of critical micelle concentration	S14
VIII.	Characterization of BKM120-loaded nanoparticles	S15
IX.	Buffer stability of HE_{12}-SNAs	S19
X.	Nuclease resistance studies	S24
XI.	Cellular uptake studies of HE_{12}-SNAs	S26
XII.	*In vitro* cell studies	S31
XIII.	HSA binding studies	S35
XIV.	*In vivo* studies	S38
XV.	References	S44

I. General

Tris(hydroxymethyl)aminomethane (Tris), ethylenediaminetetraacetate (EDTA), urea, 40% acrylamide/bis-acrylamide (19:1), ammonium persulfate (APS), N,N,N',N'-tetramethylethane-1,2-diamine (TEMED) and agarose were purchased from BioShop Canada Inc and used without further purification. Magnesium acetate and Nile Red were purchased from Sigma-Aldrich. Acetic acid, boric acid, ammonium hydroxide and 10x DPBS (with magnesium, calcium) were purchased from Fischer Scientific and used without further purification. Acetone ACS grade was purchased from Fischer. GelRed^{TM} nucleic acid stain was purchased from Biotium Inc. GeneRuler DNA Ladder Mix and DNA Gel Loading Dye (6X) were obtained from Thermo Scientific. 1 µmole 1000 Å universal synthesis CPG column, standard reagents used for automated DNA synthesis and Sephadex G25 (super fine DNA grade) were purchased from BioAutomation. DMT-1,12-dodecane-diol (HE, cat.# CLP-1114) phosphoramidites was purchased from ChemGenes corporated. 1x TBE buffer is composed of 90 mM Tris, 90 mM boric acid and 2 mM EDTA with a pH ~8.3. 1x TAMg buffer is composed of 45 mM Tris, 20 mM acetic acid and 12.5 mM $Mg(oAc)_2{\cdot}4H2O$, and its pH was adjusted to ~8.0 using glacial acetic acid. 1x DPBS (with magnesium and calcium) is composed of 8 mM sodium phosphate dibasic, 138 mM of sodium chloride, 1.47mM of potassium phosphate monobasic, 2.6 mM potassium chloride, 0.5 mM magnesium chloride (anhydrous) and 0.9 mM calcium chloride (anhydrous).

II. Instrumentation

Standard oligonucleotide synthesis was performed on solid supports using a Mermade MM6 synthesizer from Bioautomation. HPLC purification was carried out on an Agilent Infinity 1260. UV absorbance DNA quantification measurements were performed with a NanoDrop Lite spectrophotometer from Thermo Scientific. For structure assembly, Eppendorf Mastercycler 96-well thermocycler and Bio-Rad T100TM thermal cycler were used to anneal all DNA nanoparticles. Polyacrylamide gel electrophoresis (PAGE) was performed using 20x20 cm vertical Hoefer 600 electrophoresis units. Agarose Gel Electrophoresis (AGE) were performed on Owl Mini and Owl EasyCast horizontal gel systems. Gels were imaged by BioRad ChemiDoc MP system. Equilibrium dialysis was performed using single-use DispoEquilibrium Dialyzers (5000 Dalton molecular weight cut-off) from Harvard Apparatus. Fluorescence data were measured by BioTek Synergy H4 Hybrid Multi-Mode Microplate Reader. Multimode 8 scanning probe microscope and Nanoscope V controller (Bruker, Santa Barbara, CA) was used to acquire AFM images. DynaPro (model MS) molecular-sizing instrument was used to measure the particle size distributions. Liquid Chromatography Electrospray Ionization Mass Spectrometry (LC-ESI-MS) was carried out using a Bruker MaXis Impact™. TEM micrographs were acquired on FEI Tecnai 120 kV 12 microscope (FEI electron optics). Fluorescence cell imaging was performed with a Zeiss Axio Imager. Cytotoxicity studies were performed using the CellTiter96 kit from Promega according to the manufacturer's instructions. Apoptosis studies were analysed using a FACS Calibur flow cytometer. *In vivo* fluorescence measurements were performed using In Vivo Imaging System (IVIS).

III. Solid-phase synthesis, purification

DNA synthesis was performed on a 1 μmole scale, starting from a universal 1000 Å LCAA-CPG solid-support.[1] Coupling efficiency was monitored after removal of the dimethoxytrityl (DMT) 5′-OH protecting groups. DMT-dodecane-diol phosphoramidite (cat.# CLP-1114) was purchased from ChemGenes. Cyanine 3 Phosphoramidite (cat.# 10-5913-02) and Cy5.5 phosphoramidite (cat# 10-5961-95) were purchased from Glen Research. DMT-dodecane-diol and Cy3 phosphoramidite were dissolved in the acetonitrile under a nitrogen atmosphere in a glove box (<0.04 ppm oxygen and <0.5 ppm trace moisture). For DMT-dodecane-diol (0.1M, anhydrous acetonitrile) and Cy3 (0.1M, anhydrous acetonitrile) amidites, extended coupling times of 5 minutes were used respectively using 0.25M 5-(ethylthio)tetrazole in anhydrous acetonitrile. The Cy3 addition was performed under a nitrogen atmosphere in a glove box. Removal of the DMT protecting group was carried out using 3% dichloroacetic acid in dichloromethane. Completed syntheses were cleaved from the solid support and deprotected in 28% aqueous ammonium hydroxide solution for 16-18 hours at 60°C. In the case of Cy5.5, the mixture was deprotected in 28% aqueous ammonium hydroxide solution for 24-36 hours at room temperature. Following deprotection, the crude solid was re-suspended in 1 mL Millipore water and passed through a 0.22 μm centrifugal filter prior to HPLC purification. The resulting solution was quantified by absorbance at 260 nm. For HPLC purification, solvents (0.22 μm filtered): 50mM Triethylammonium acetate (TEAA) buffer (pH 8.0) and HPLC grade acetonitrile. Elution gradient: 3-70% acetonitrile over 30 minutes at 60°C. Column: Hamilton PRP-C18 5 μm 100 Å 2.1 x 150 mm. For each analytical separation, approximately 0.5 OD_{260} of crude DNA was injected as a 20-100 μL solution in Millipore water. Detection was carried out using a diode-array detector, monitoring absorbance at 260 nm. Retention times and for the products are summarized in Table ST2.

Alternatively, in the case of ssDNA, the crude product was isolated, dried, and re-suspended in 1:1 H_2O/8 M urea before loading to 20% polyacrylamide/urea gel. The gel was run at 250 V for 30 minutes followed by 500 V for 60 minutes with 1x TBE as the running buffer. The gel was then imaged and excised on TLC plate under a UV lamp. DNA was extracted from the excised gel slabs by crushing and soaking in 11-12 mL Milli-Q water at 60°C overnight. The solution was dried to approximately 1 mL before loading to Sephadex G-25 column. The purified DNA was quantified by its absorbance at 260 nm.

IV. Sequences of DNA-polymer conjugates and characterization

The sequences of the DNA-polymer conjugates and DNA controls are presented in Supporting Table 1 (ST1).[2]

Supplementary Table ST1| Sequences used for DNA amphiphiles and DNA controls. (**D** = DMT-dodecane-diol), (**Cy3**= Cyanine 3 phosphoramidite), (**Cy5.5**= Cyanine 5.5 phosphoramidite).

Strand	Sequence (5'-xx-3')
**HE_{12}-DNA**	**DDDDDDDDDDDD**TTTTTCAGTTGACCATATA
**Cy3-HE_{12}-DNA**	**Cy3DDDDDDDDDDDD**TTTTTCAGTTGACCATATA
**Cy5.5-HE_{12}-DNA**	**Cy5.5DDDDDDDDDDDD**TTTTTCAGTTGACCATATA
**ssDNA**	TTTTTCAGTTGACCATATA
**Cy5.5-ssDNA**	**Cy5.5**TTTTTCAGTTGACCATATA
**Cy3-ssDNA**	**Cy3**TTTTTCAGTTGACCATATA

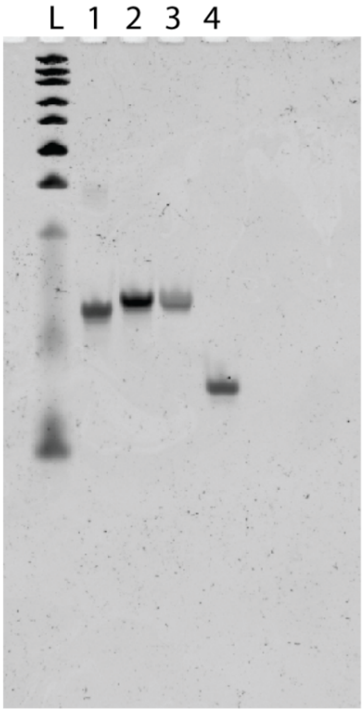

Supplementary Figure 1. Denaturing gel electrophoresis of the DNA-polymer conjugates. (18% denaturing PAGE). L: ladder, lane 1: HE_{12}-DNA, Lane 2: Cy3-HE_{12}-DNA, Lane 3: Cy5.5-HE_{12}-DNA, Lane 4: ssDNA control

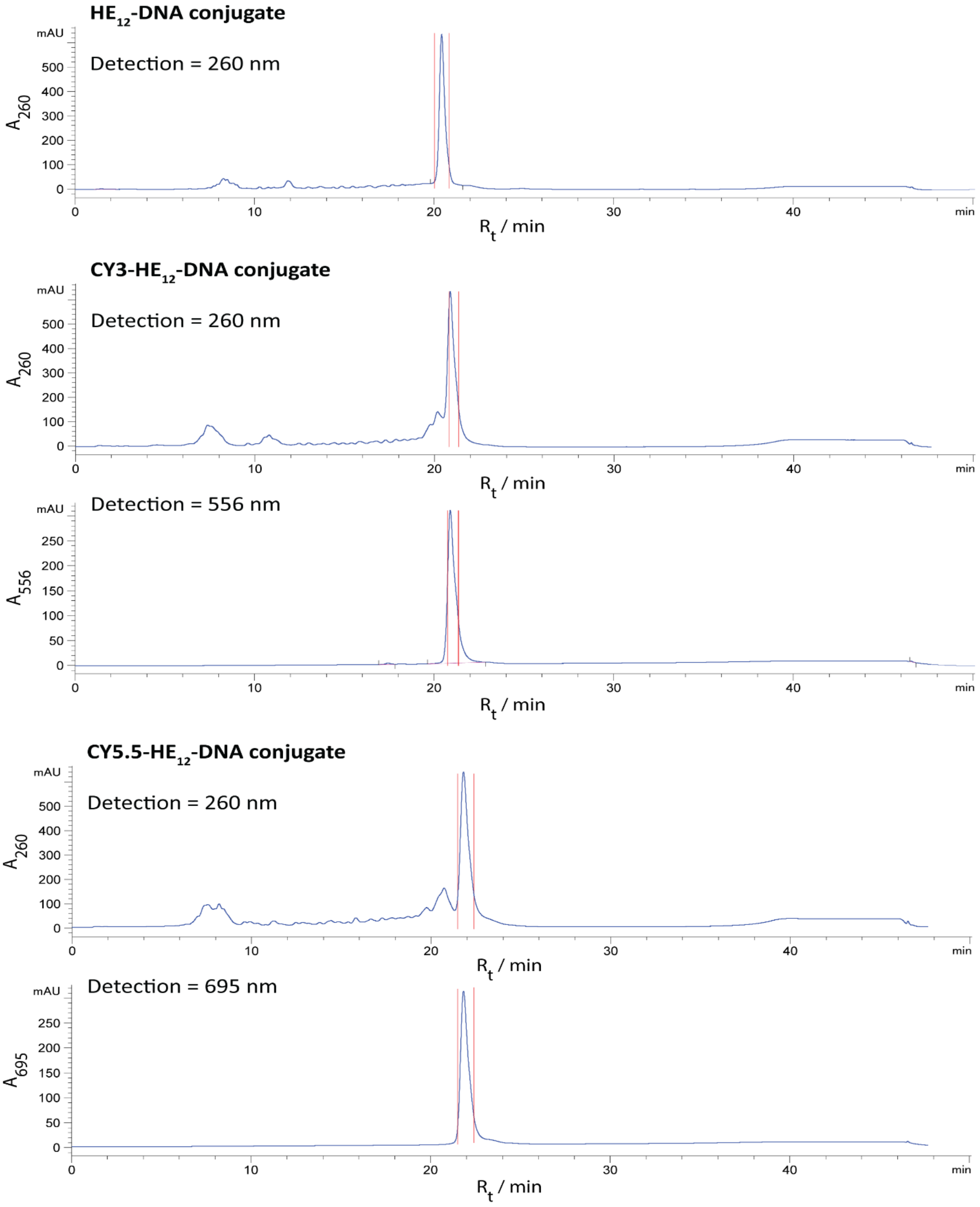

Supplementary Figure 2. HPLC chromatograms of the crude DNA-polymer products measured at 260 nm, 556 nm (Cy3-specific) and 695 nm (Cy5.5-specific). Elution gradient: 3-70% acetonitrile over 30 minutes at 60°C.

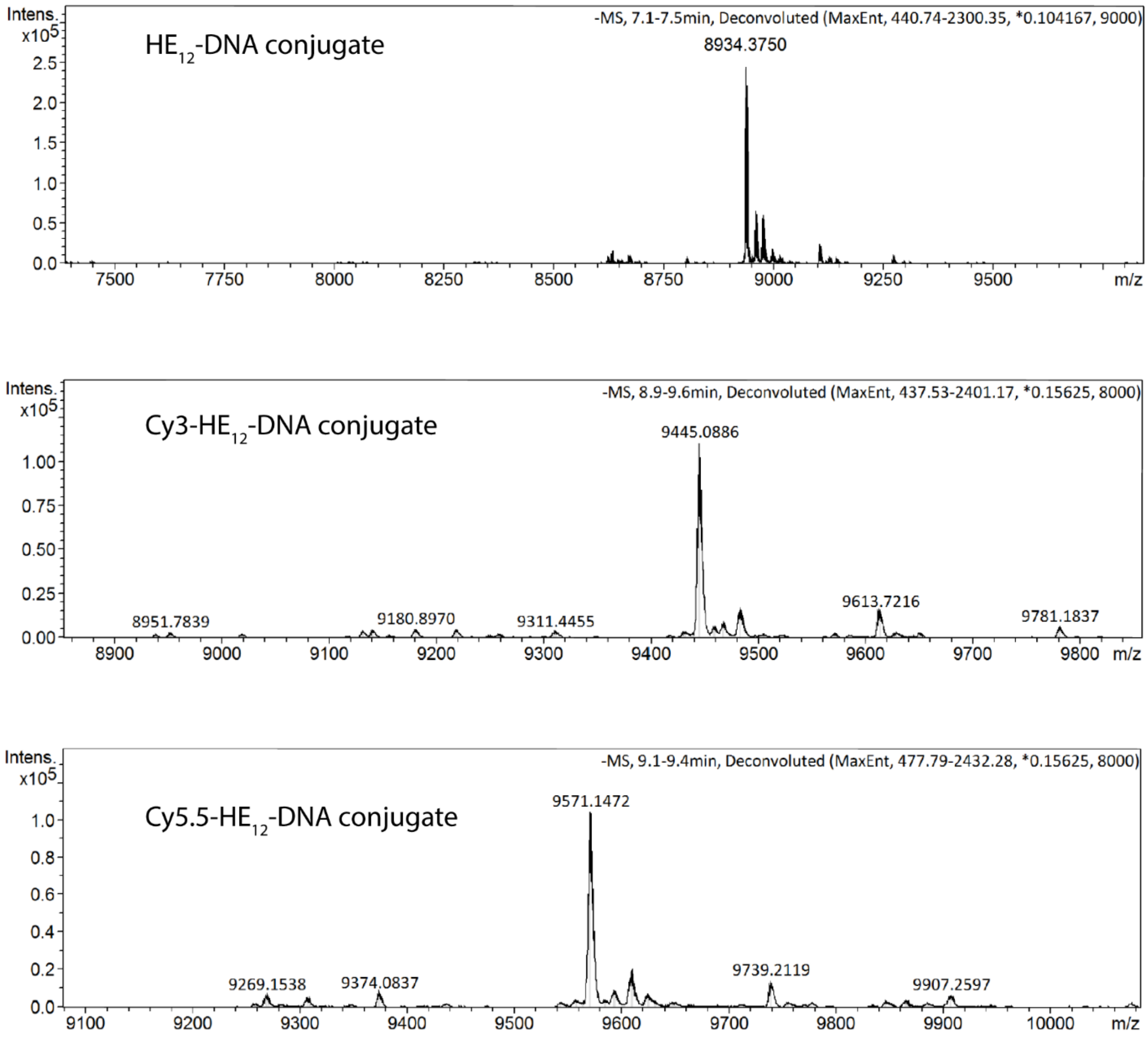

Supplementary Figure 3. MS characterization of DNA-polymer conjugates.

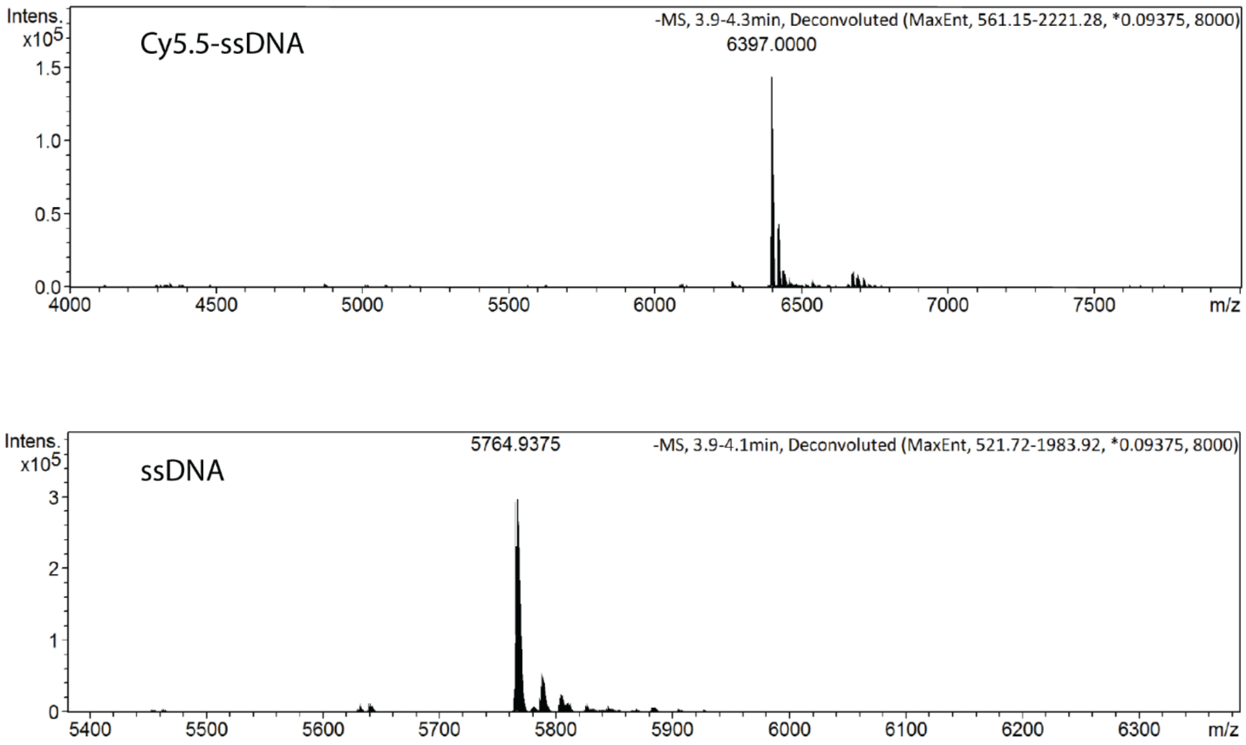

Supplementary Figure 4. MS characterization of ssDNA controls.

Supplementary Table ST2| LC-ESI-MS Calculated and experimental m/z values for all DNA amphiphiles synthesized including the unmodified oligonucleotide controls.

Molecule	Calculated m/z	Found m/z
**HE_{12}-DNA**	8933.77	8934.3750
**Cy3-HE_{12}-DNA**	9442.02	9445.0886
**Cy5.5-HE_{12}-DNA**	9568.07	9571.1472
**ssDNA**	5764.99	5764.9375
**Cy5.5-ssDNA**	6398.28	6397.000

V. Evaluation of the encapsulation of BKM120:

BKM120 was prepared as a 10 mM working solution in ethanol. Loading of the structures was achieved by adding 10 µL of BKM120 to an eppendorf, followed by solvent evaporation in open air to achieve a thin drug film. HE_{12}-DNA conjugates in water were then added the drug film, mixed and followed by the addition of the assembly buffer (final solution: 100 µL at 10 mM in 1xTAMg buffer). The final solution was 100 µL with 100x excess BKM120 (1 mM). The mixture was vortexed heavily to allow re-suspension of the drug molecules, and was then annealed at 95°C to 10°C over 4 hours. Following the annealing step, removal of free BKM120 was achieved by preparative microcentrifugation (2 cycles of 14,000 rpm for 25 minutes, 4°C) to remove excess drug precipitate. Following microcentrifugation, the mixture was then purified by size-exclusion chromatography using Illustra MicroSpin G-25 Columns (GE Healthcare) using a modified protocol. (The columns were washed twice before resuspension in 1x TAMg. For the elution step, the spinning time was also optimized to ensure higher yield of recovered DNA nanoparticles).

a) BKM120 loading capacity of HE_{12}-SNAs:

Reversed phase-HPLC was used to determine the amount of BKM120 loaded in the DNA nanoparticles, For HPLC, 60 µL of the purified supernatant was injected into a Hamilton PRP-1 5µm 100 Å 2.1 x 150mm column. The solvents used are 50 mM triethylammonium acetate (TEAA) buffer (pH 7.8) and HPLC grade acetonitrile. Typical retention times for the products are 27.4 minutes (DNA-polymer conjugate) and 28.733 minutes (BKM120) at 260 nm detection channel. The products were also detected using a drug-only channel at 320 nm (BKM120 maximum absorption peak). BKM120 loading capacity was determined by measuring the DNA peak areas at 260 nm and drug peak area 320 nm (see Figure SF3). The values were compared with known concentration standards to obtain the number of drug molecules/DNA-conjugate strand. The loading capacity was also calculated based on the equation below:

Loading content (LC) % = mass of BKM120 in nanoparticles / total mass of loaded nanoparticles x 100%

The percent yield was calculated from the peak area of the recovered DNA product using known standards, and comparing it to initial starting concentration of 10 µM.

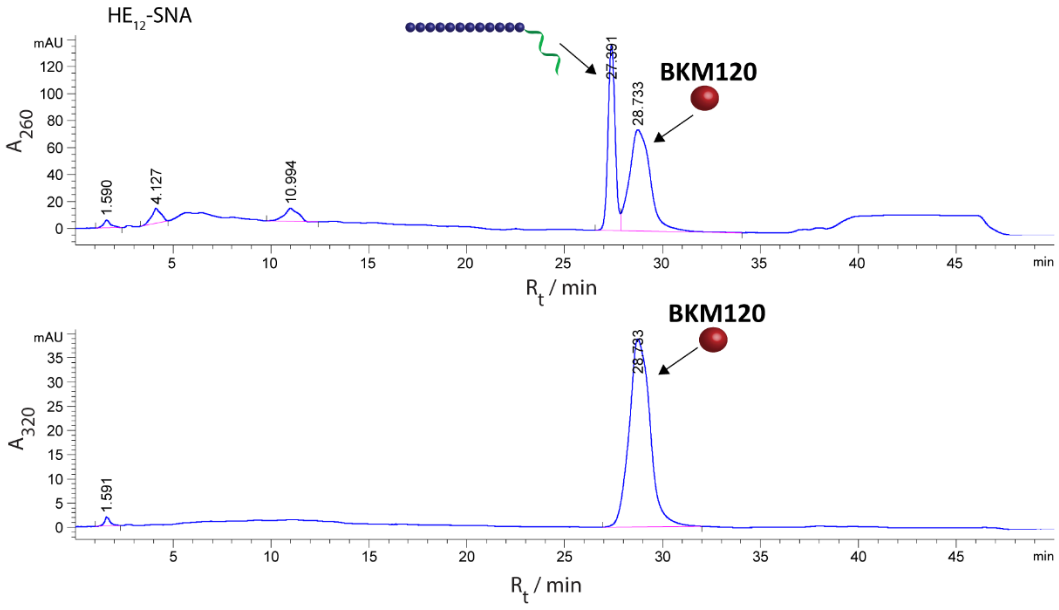

Signal detection: 260 nm		
**Peak**	**Retention time [min]**	**Average Area (mAU*s)**
HE_{12}-DNA	27.391	3687.33
BKM120	28.733	3168.43

Signal detection: 320 nm		
**Peak**	**Retention time [min]**	**Average Area (mAU*s)**
BKM120	28.733	2868.46

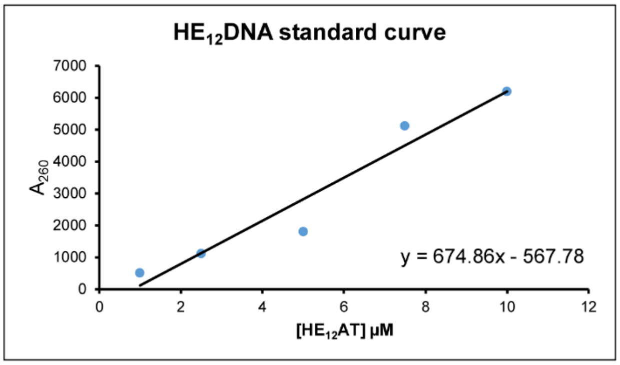

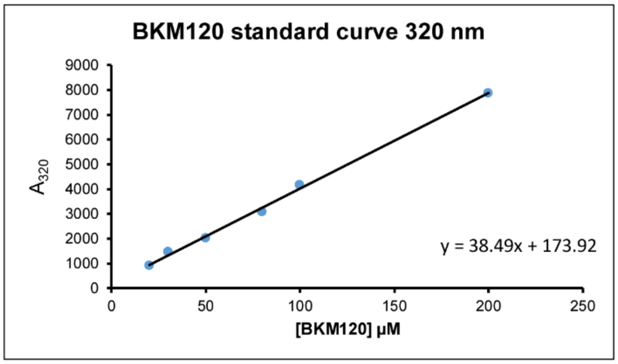

Sample	Calculated [DNA] µM	Yield [%]	Calculated [BKM120] µM	#BKM120 molecules/strand
HE_{12}-SNA	6.3	~ 65	59.5	~ 9

Supporting Figure 5. BKM120 loading capacity in HE_{12}-SNAs calculated from RP-HPLC data.

b) UV-Vis spectroscopy

UV-vis measurements were used to ensure BKM120 encapsulation following the purification method. For each measurement, 100 µL of each of the purified solutions were dropped on a 96-well plate reader and measured through a BioTek Synergy H4 Hybrid Multi-Mode Microplate Reader. Data from UV-Vis spectroscopy was used to confirm the calculated drug concentration from RP-HPLC.

c) BKM120 release kinetics

BKM120 release was evaluated by monitoring the decrease in concentration of the drug from a solution of loaded nanoparticles dialyzed against 1x TAMg buffer at room temperature over 24 hrs. Immediately following purification, the stock solution was divided into 50 µL aliquots which were dialyzed against 50 µL 1x TAMg buffer using single use DispoEquilibrium Dialyzers (5000 Dalton molecular weight cut-off) from Harvard Apparatus. The samples separated into different tubes and incubated at room temperature, then collected at each timepoint to be analyzed by RP-HPLC. Drug release was assessed by the decrease of drug concentration from the chamber containing the DNA nanoparticles. The amount of DNA remained constant in each measured indicating no loss of structures throughout the dialysis process. One limitation of the method, however, is the low volume dialyzed from the buffer chamber. This could result in a plateau of drug release from the DNA structures as the buffer solution reaches drug saturation over time.

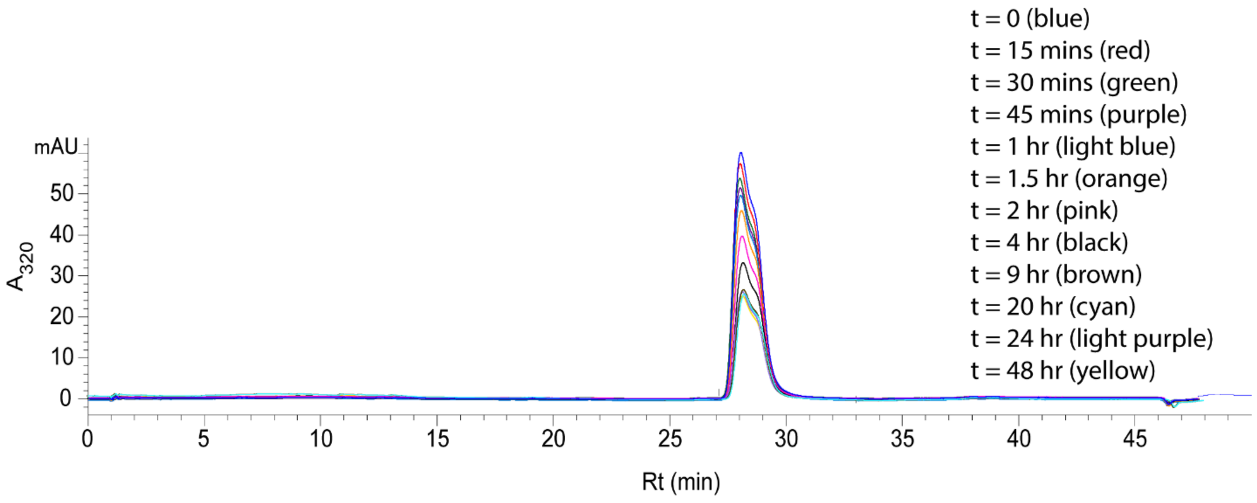

Supporting Figure 6. HPLC traces of collected dialysis fractions showing BKM120 release. The samples were detected at a BKM120-specific channel at 320 nm.

VI. Shelf-life of BKM120-loaded DNA nanoparticles:

The shelf-life of BKM120-loaded DNA nanoparticles was assessed by dynamic light scattering (DLS). The structures were stored at 4°C and room temperature, and the hydrodynamic radius was measured over a 4-week period.

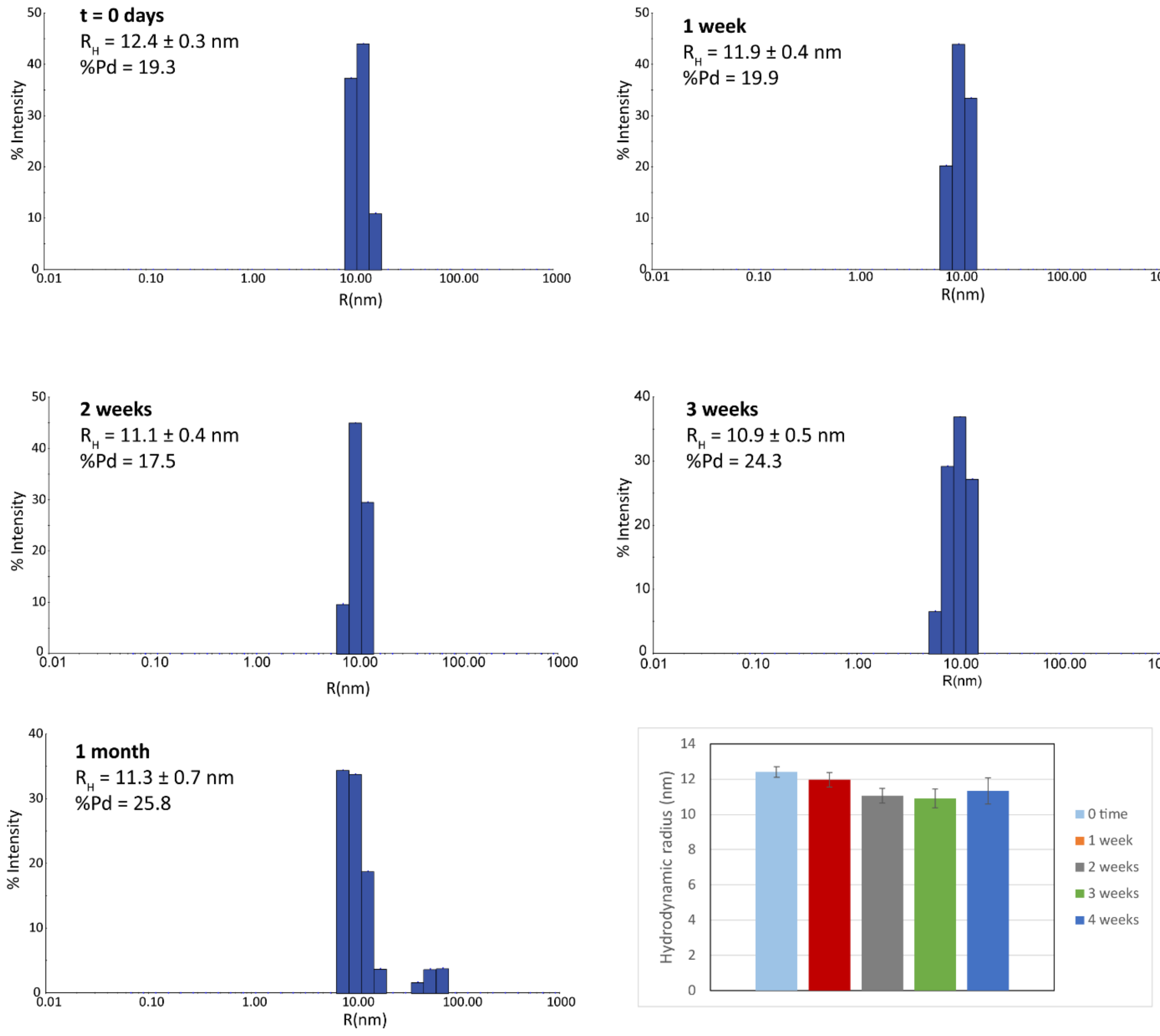

Supporting Figure 7. Shelf-life of BKM120-loaded DNA nanoparticles stored at 4°C. Dynamic light scattering (DLS) histograms showing the hydrodynamic radius of BKM120-loaded nanoparticles over time when stored at 4°C.

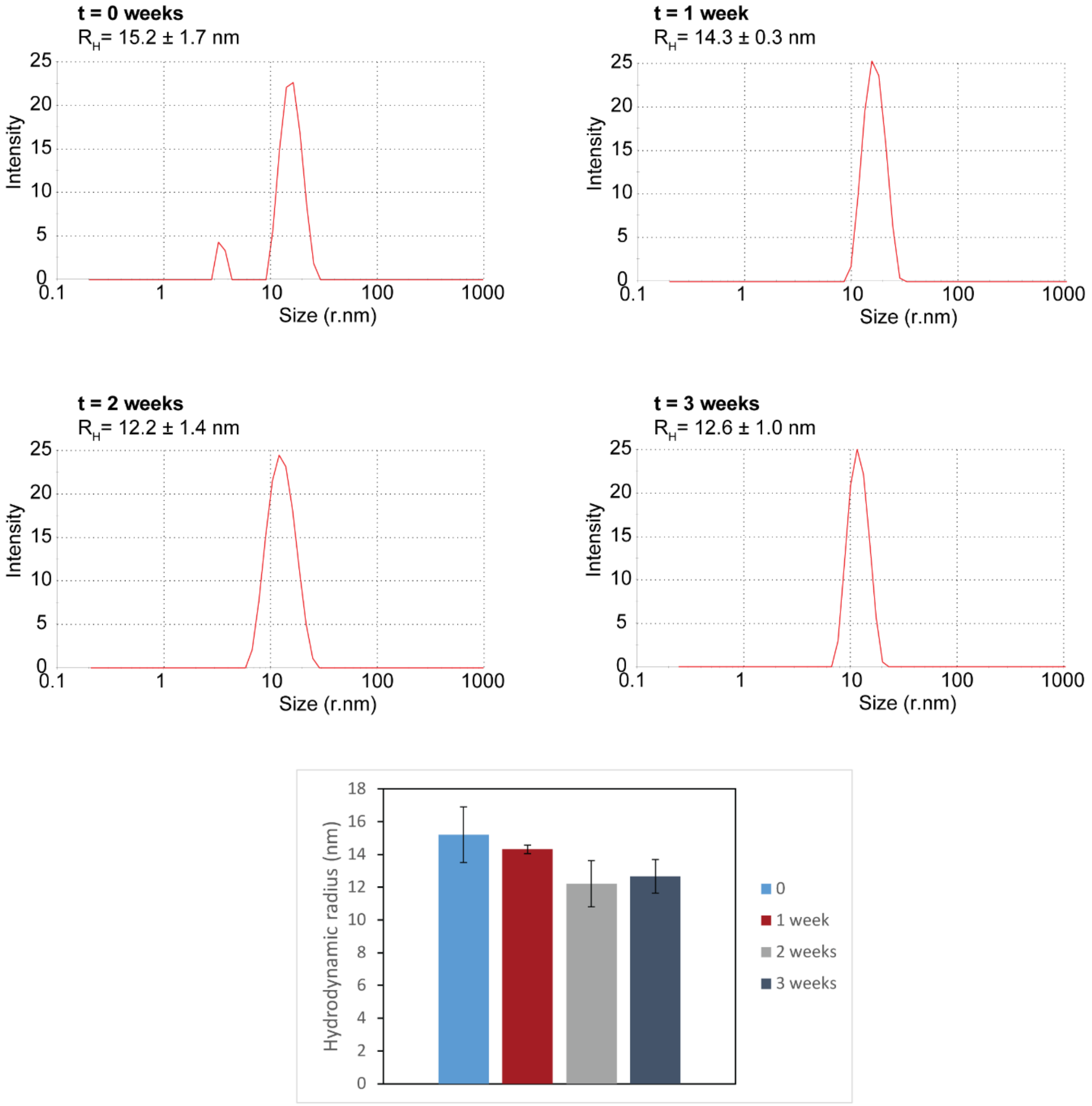

Supporting Figure 8. Shelf-life of BKM120-loaded DNA nanoparticles stored at room temperature. Dynamic light scattering (DLS) histograms showing the hydrodynamic radius of BKM120-loaded particles over time when stored at room temperature.

VII. Determination of critical micelle concentration (CMC)

To determine the CMC of HE_{12}-DNA polymer conjugates, fluorescence spectra of 100 µM Nile Red in tris-acetate-magnesium (1 x TAMg) buffer were measured in the presence of increasing concentrations of HE_{12}-DNA polymer conjugates. A stock solution of Nile Red 1 mM in acetone was used for all experiments. 1 µL of Nile Red stock in acetone was added and briefly incubated at room temperature to allow solvent evaporation. Series dilutions of DNA-polymer conjugates (in the range of 50 nM to 10 µM) were made up to a final volume of 100 µL. The mixture was subjected to a heat-cool cycle (95° C - 4° C, over 4 hours). The samples were then transferred to a 96-well top-read microplate, and the plate was read using a Bioteck Synergy well-plate fluorimeter. Excitation was at 535 nm with a slit-width of 9 nm and emission was monitored between 560 nm and 750 nm. The CMC of HE_{12}-DNA conjugates was investigated using fluorescence emission of a hydrophobic dye, Nile Red. This molecule is nearly non-emissive in bulk aqueous media, but its inclusive in a nonpolar microenvironment such as the core of HE_{12}-SNAs results in an intense fluorescence signal.[3] A CMC of 0.5 µM ± 0.2 µM was calculated for HE_{12}-DNA conjugates in 12.5 mM Mg^{2+}. We anticipate the CMC to further decrease in the BKM120-loaded structures upon the encapsulation of the drug due to the additional stabilizing π-π interactions between drug molecules, and van der walls interactions with the carbon chains of the hydrophobic core.

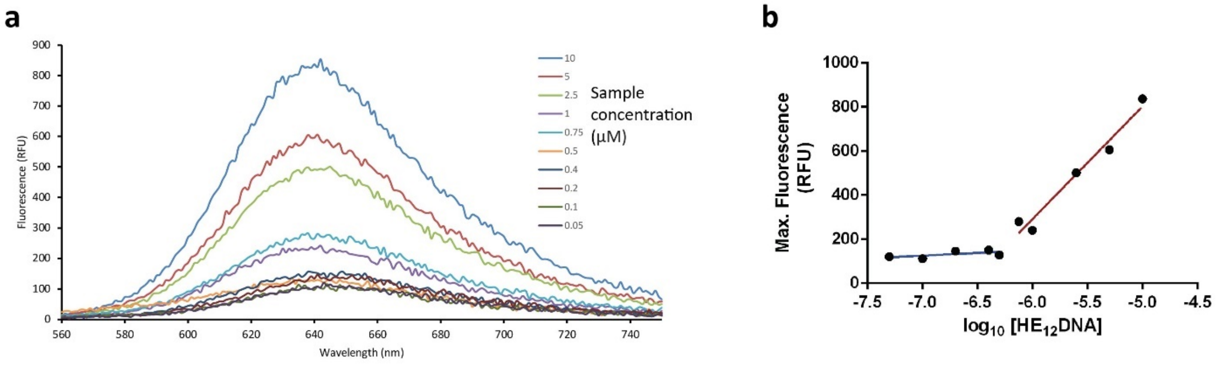

Supporting Figure 9. Fluorescence spectra of Nile Red encapsulation for determination of critical micelle concentration (CMC). a) Fluorescence spectra of Nile Red with varying concentration of DNA. b) Plot of $\log_{10}$ [HE_{12}-DNA] against maximal fluorescence intensity for HE_{12}-DNA in the presence of 100 µM Nile Red. The CMC was calculated from the intersection of the two linear fits shown on the graph. The measurements were performed in triplicates.

VIII. Characterization of BKM120-loaded DNA nanoparticles

Gel Mobility Shift Assays

Agarose gel electrophoresis was used to characterize the BKM120-loaded HE_{12}-SNAs. In each case, 2.5% AGE was carried out at 4°C for 2.5 hours at a constant voltage of 80V. Typical sample loading is 15 picomoles with respect to the DNA, per lane (1.5 µL of 10 µM DNA).

Dynamic Light Scattering

Dynamic light scattering (DLS) experiments were carried out using a DynaPro™ Instrument from Wyatt Technology. A cumulants fit model was used to confirm the presence and determine the size the BKM120-loaded HE_{12}-SNAs. Sterile water and 1xTAMg buffer were filtered using a 0.45 µm nylon syringe filter before use in DLS sample preparation. 20 µL of sample (concentration: 10 µM) was used in each measurement. All measurements were carried out in triplicate at 25°C.

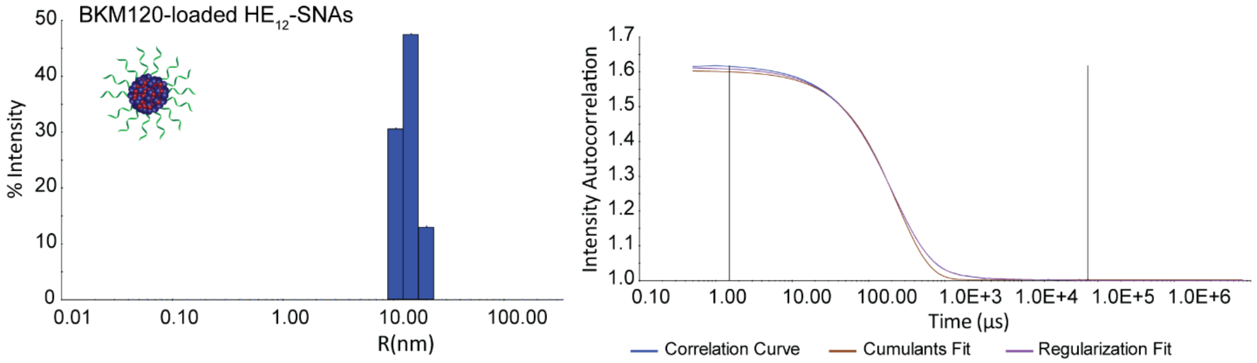

Supporting Figure 10. Additional DLS results for BKM120-loaded HE_{12}-DNA nanoparticles. Left: histogram showing the size distribution of the structure; right: the intensity correlation function. The structures show a hydrodynamic radius R_H = 11.8 ± 0.4 nm.

Atomic Force Microscopy

Dry AFM was carried out using a MultiMode8™ SPM connected to a Nanoscope™ V controller (Bruker, Santa Barbara, CA). All images were obtained using ScanAsyst mode in air with AC160TS cantilevers (Nominal values: Tip radius – 9 nm, Resonant frequency – 300 kHz, Spring constant – 42 N/m) from Asylum Research. Samples were diluted to 1 μM in TAMg buffer and 4μL of this solution was deposited on a freshly cleaved mica surface (ca. 7 x 7 mm) and allowed to adsorb for 1-2 seconds. Then 50 μL of 0.22 μm filtered Millipore water was dropped on the surface and instantly removed with filter paper. The surface was then washed with a further 200 μL of water and the excess removed with a strong flow of nitrogen. Samples were dried under vacuum for 15-30 minutes prior to imaging.

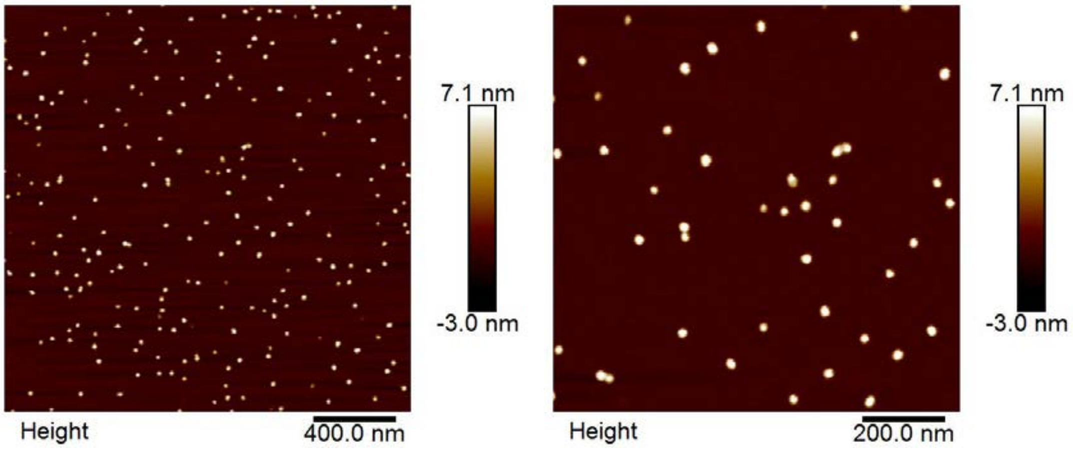

Supporting Figure 11. Additional AFM images of BKM120-loaded HE_{12}-DNA nanoparticles. Following purification, spherical drug-containing particles are recovered. Average size of the particles was 27.8 ± 4.3 nm, with height of 8.1 ± 0.9 nm (N = 43).

Transmission Electron Microscopy

Samples (2 μL at 0.5 μM w.r.t. total DNA) were deposited on carbon film coated copper EM grids for one minute, followed by blotting off the excess liquid with the edge of a filter paper, and washing three times with 20 μL of water, before drying under vacuum. The samples were imaged using a Tecnai 12 microscope (FEI electron optics) equipped with a Lab6 filament at 120 kV. Images were acquired using a Gatan 792 Bioscan 1k x 1k Wide Angle Multiscan CCD Camera (Gatan Inc.). Contrast was adjusted automatically - note that in the presence of any high-contrast foreign matter, the structures resulted in being almost invisible. Images were analyzed using ImageJ, which required manually setting threshold levels and placing limits on the size and circularity of features to ensure correct particle picking. The area values obtained were converted into radii (for comparison with DLS), making the assumption that the features are circular, which can be readily validated by eye.

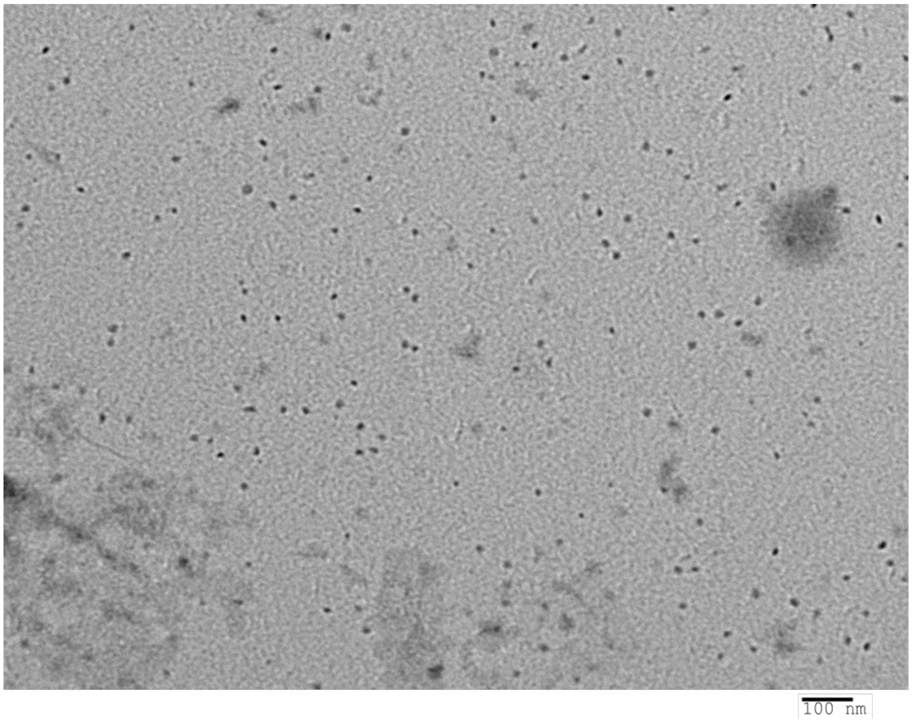

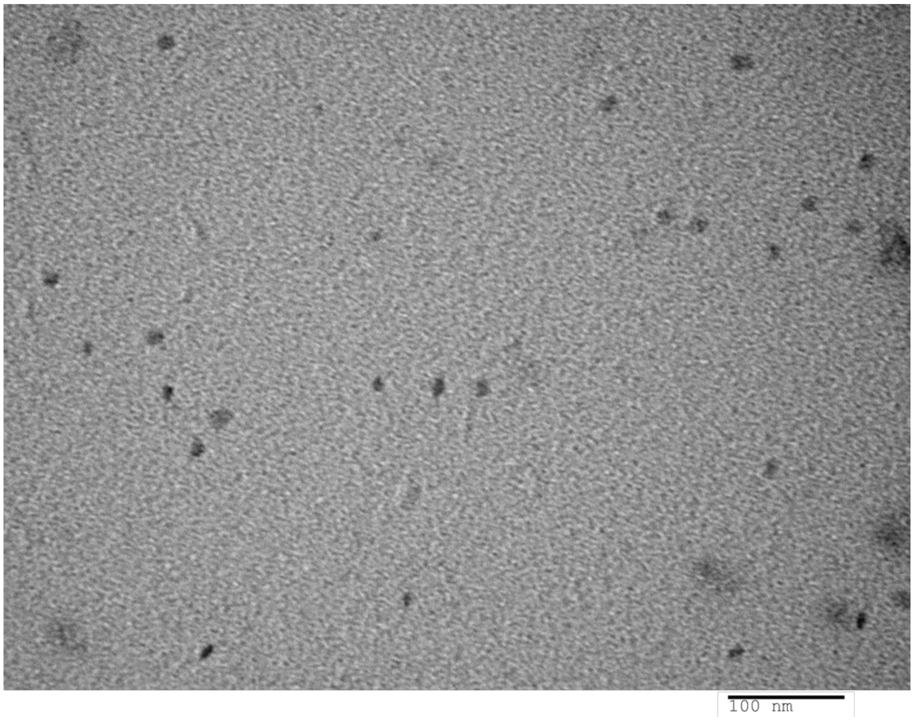

Supporting Figure 12. Additional TEM images of BKM120-loaded HE_{12}-SNAs. Average diameter was calculated to be 21 ± 3 nm (N = 56).

IX. Buffer stability of HE_{12}-SNAs

The stability of HE_{12}-SNAs in buffer was evaluated by DLS measurements. Tris buffer containing different concentrations of magnesium or calcium was added to HE_{12}-DNA conjugates and the mixture was annealed 95°C-4°C over 4 hours. In the case of DPBS buffer, different amounts of DPBS buffer were diluted from an initial 10X stock, mixed with HE_{12}-DNA conjugates in water and annealed 95°C-4°C over 4 hours, prior to DLS analysis.

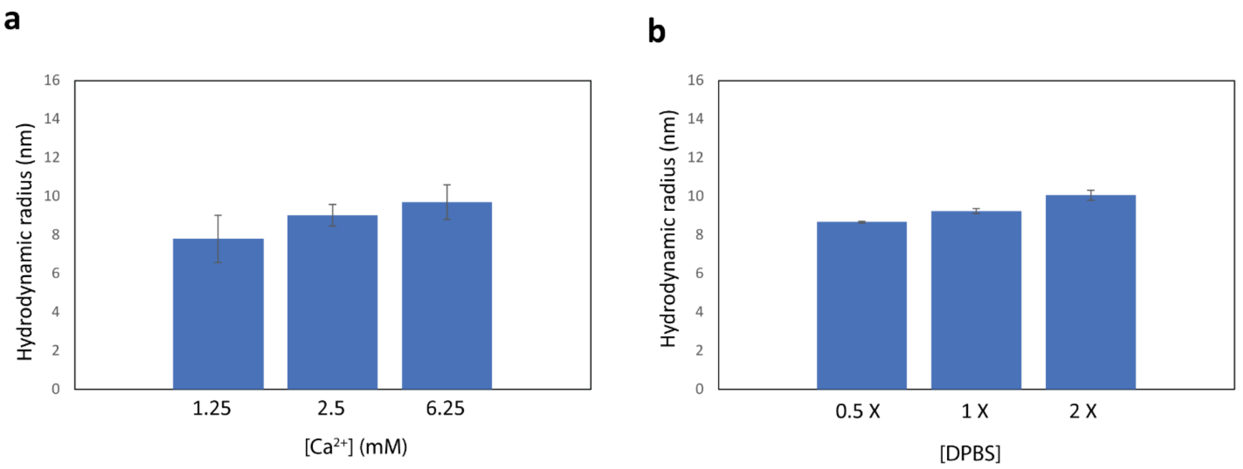

Supporting Figure 13. Stability of HE_{12}-SNAs under biological relevant conditions. DLS histograms displaying the hydrodynamic radius of DNA nanoparticles under variations in a) calcium concentrations in Tris buffer; b) with varying amounts of DPBS, a cell culture buffer. DLS analysis show the maintained structural integrity of HE_{12}-DNA particles under large variations of ionic conditions.

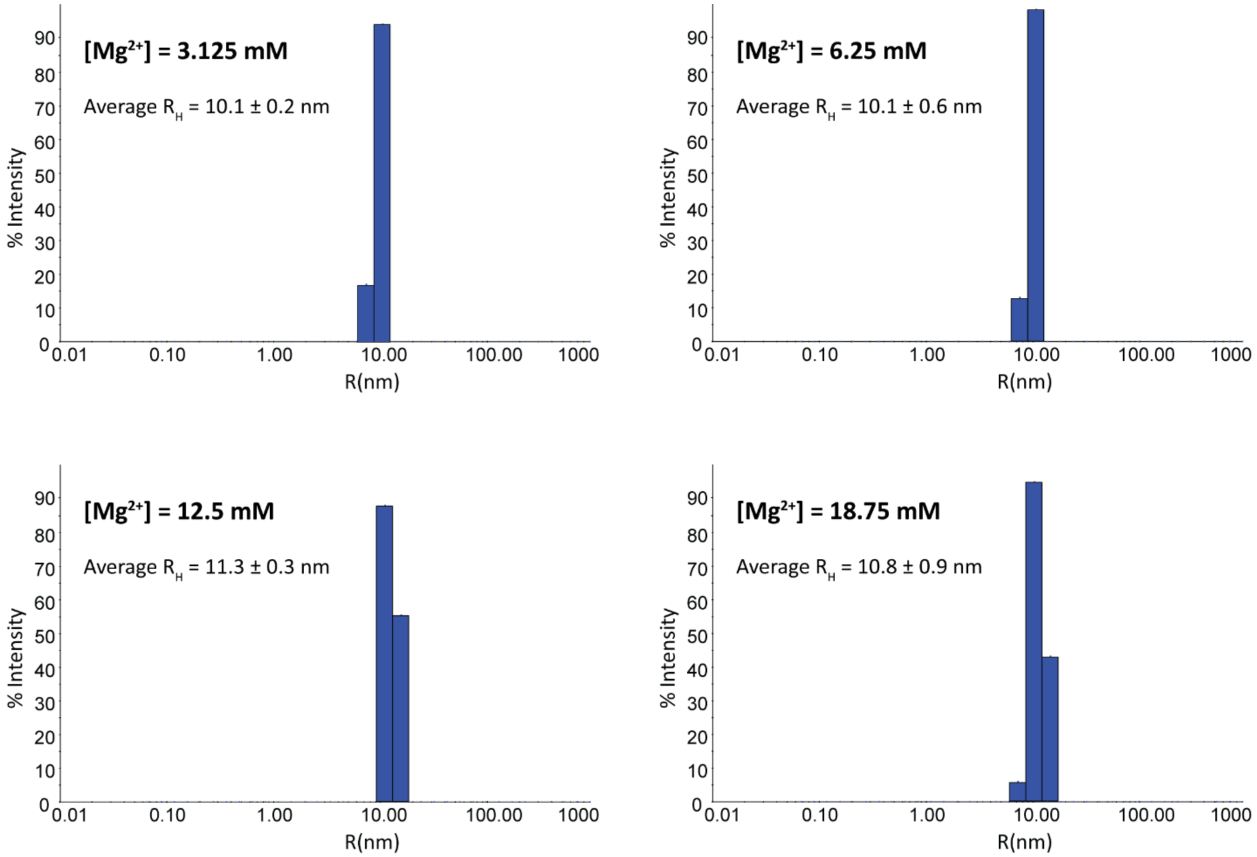

Supporting Figure 14. DLS histograms of HE_{12}-SNAs under different magnesium concentrations in Tris buffer.

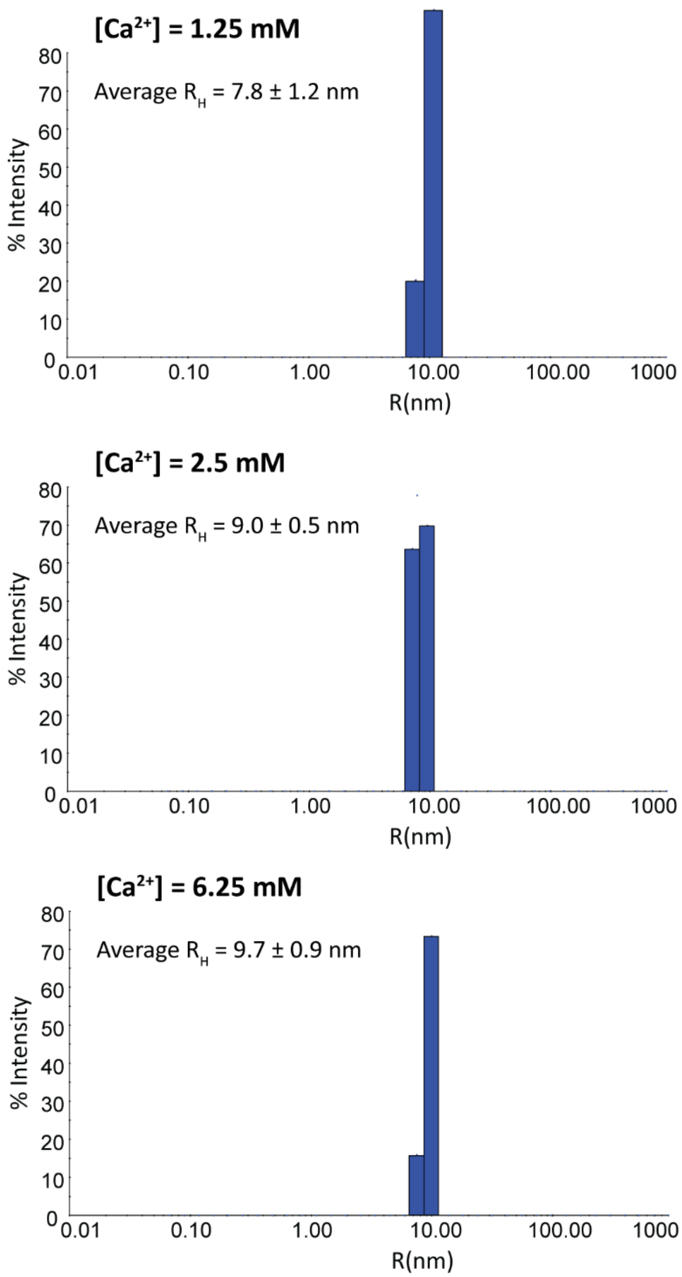

Supporting Figure 15. DLS histograms of HE_{12}-SNAs under different calcium concentrations in Tris buffer.

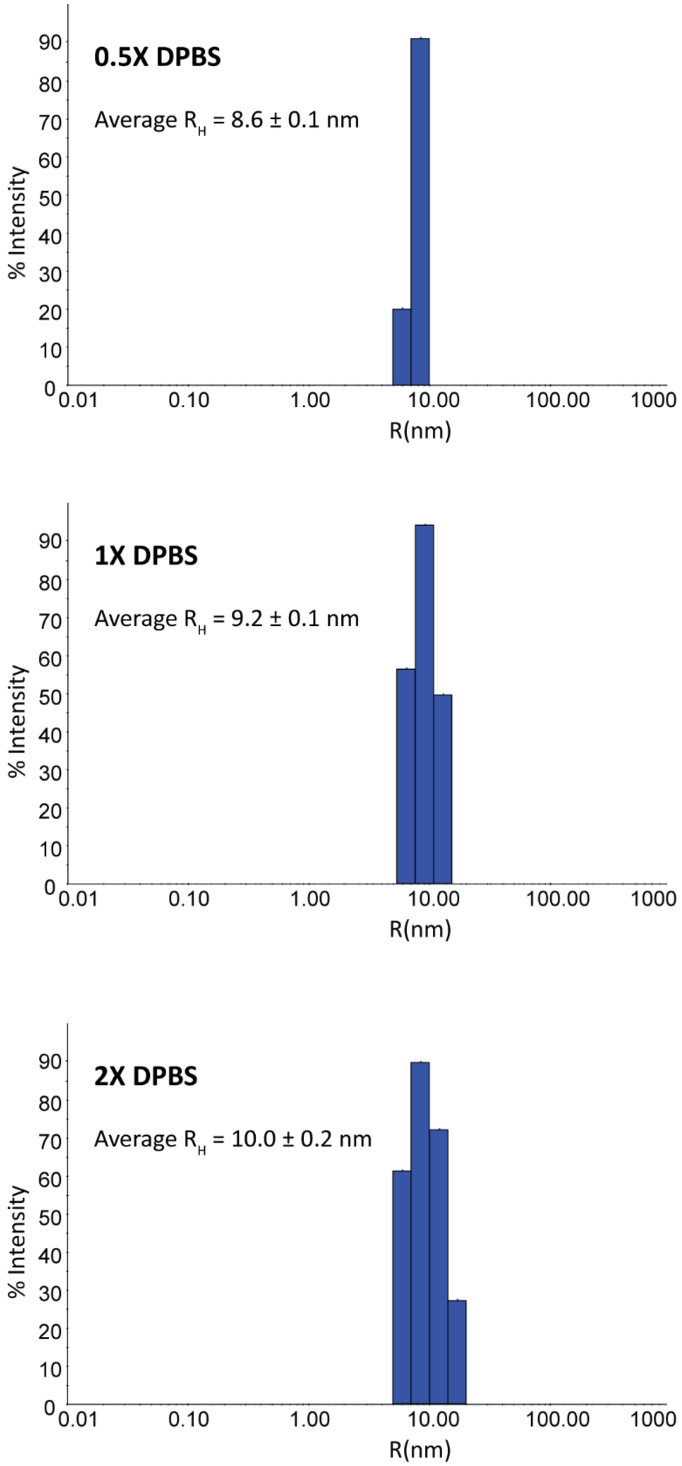

Supporting Figure 16. DLS histograms of HE_{12}-SNAs under different amounts of DPBS buffer.

Depletion of divalent cations causes the disassembly of structures into monomeric units. The assembly of HE_{12}-SNAs in a Tris buffer containing no divalent cations, and in water is described below.

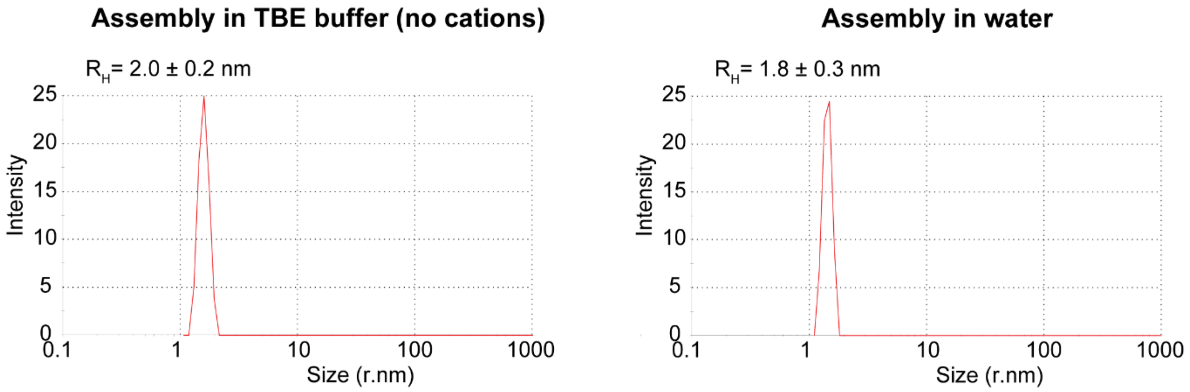

Supporting Figure 17. The effect of cation depletion on HE_{12}-SNAs. DLS histograms of HE_{12}-SNAs assembly in TBE buffer (tris buffer containing no divalent cations), and in water. A hydrodynamic radius in the range of 2 nm is indicative of monomeric HE_{12}-DNA conjugate units and the lack of higher order assembly.

X. Nuclease resistance studies

DNA nanoparticle nuclease resistance compared to single stranded DNA was measured by a nuclease degradation assay published by Conway et. al.[4] Samples (10 µM) were incubated with 10% Fetal Bovine Serum (FBS) for several time points at 37°C. At each time point an aliquot was removed and immediately added to a stop solution (Formamide 50%/SDS 0.01%), proteinase K (2 units) and stored at -20°C. Samples were then resolved on 20% PAGE (TBE) denaturing gel (20.7 mL H2O, 1.8 mL of 1x TBE, 7.5 mL 40% acrylamide, 8 M urea). Gels were stained with Gel Red (Biotium, USA) and imaged by a Biorad Imager. Quantification and data analysis was performed using the Graphpad Prism software. The intensity of the lower mobility band (undegraded structure) was quantified over time. The experiment was performed in triplicate. Half-lives were calculated by fitting a first order decay.

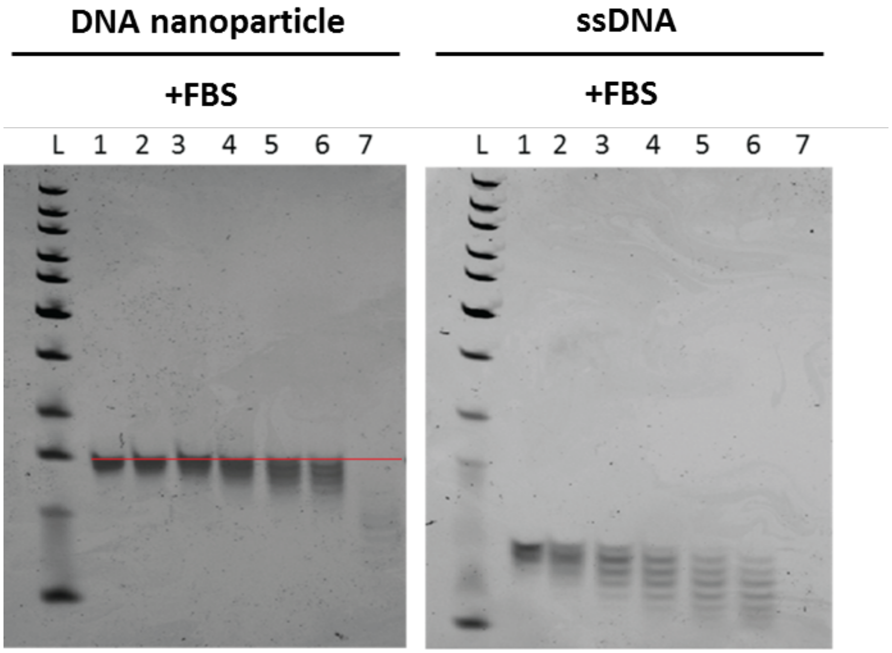

Supporting Figure 18. Denaturing PAGE gel of the FBS degradation products. L: ladder, lane 1: time 0, lane 2: 15 mins, lane 3: 30 mins, lane 4: 1 hour, lane 5: 2 hours, lane 6: 3 hours and lane 7: 24 hours. The intensity of the lower mobility band was quantified over time (red line across the gel). The experiment was performed in triplicate.

Phosphorothioated DNA nanoparticles were synthesized according to a protocol reported by Fakhoury et. al.[5] The nuclease resistance experimental conditions for phosphorothioated particles are exactly the same as unmodified structures, described above.

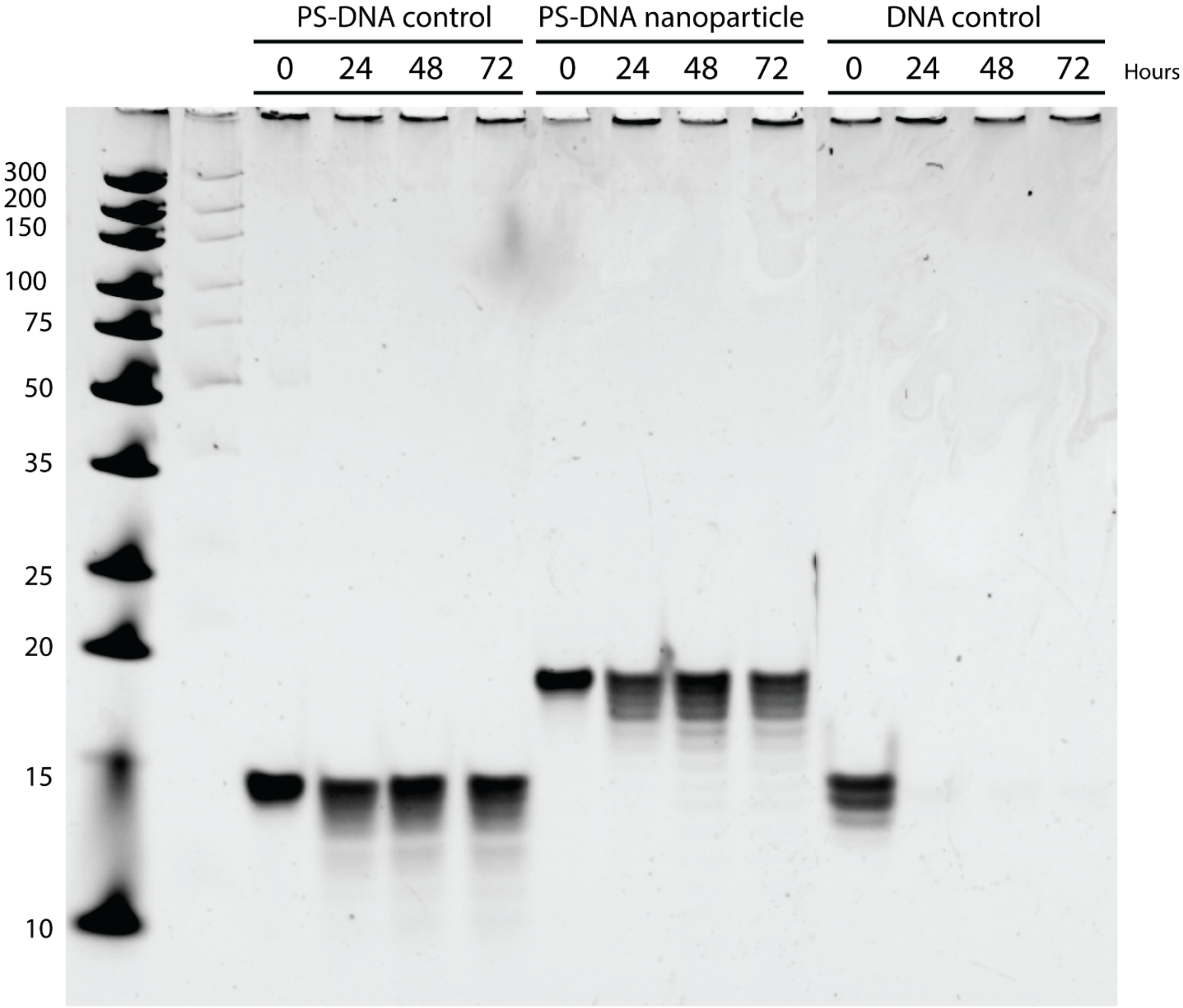

Supporting Figure 19. Denaturing PAGE gel of the FBS degradation products of phosphorothioated DNA nanoparticles. PS-DNA control: phosphorothioated DNA strand, PS-DNA nanoparticle: HE_{12}-SNA with phosphorothioated DNA, DNA control: unmodified DNA strand.

XI. Cellular uptake studies of DNA nanoparticles

Preparation of Cy3-labeled nanoparticles

Cy3-labeled HE_{12}-SNAs were prepared by mixing Cy3-labeled HE_{12}-DNA with unlabelled HE_{12}-DNA strands at a 25:75 percent ration (DNA concentration 10 µM) followed by an annealing cycle 95°C-4°C over 4 hours. This percentage of labeled/unlabeled strands was observed to give the cleanest assemblies along with high fluorescence intensity for cellular uptake studies.

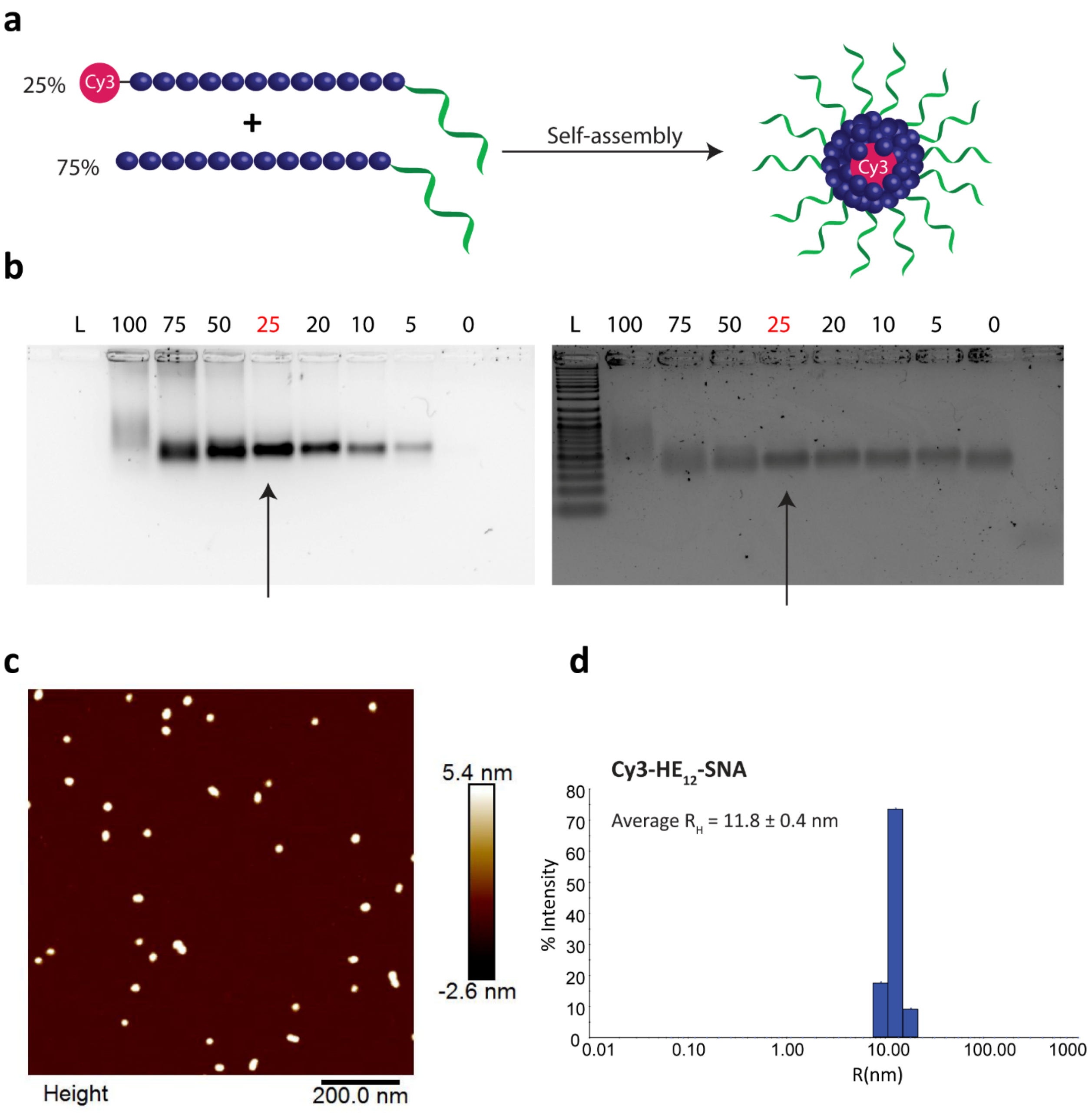

Supporting Figure 20. Preparation and characterization of Cy3-labeled HE_{12}-SNAs. a) General methodology for preparing Cy3-labeled DNA nanoparticles. b) Agarose Gel Electrophoresis (AGE) characterization of Cy3-labeled particles imaged under Cy3 channel (left) and Gel Red DNA channel (right). Numbers on lanes indicate the percentage of labeled strands in the final structure. At ratio 25:75 of Cy3-HE_{12}-DNA/unlabeled HE_{12}-DNA strands, the structures show highest fluorescence intensity and morphological integrity c) Atomic force microscopy (AFM) images of Cy3-labeled particles showing monodisperse structures with average diameter of 28.4 ± 3.6 nm. c) DLS histogram of Cy3-labeled HE_{12}-SNAs showing an in solution hydrodynamic radius of 11.8 ± 0.4 nm.

Confocal Microscopy for Cellular Uptake of Cy3-labeled nanoparticles

HeLa (adenocarcinoma) cells were seeded at a density of 5 x 10^5 in 8-well slides. After 24 hours cells were incubated with Cy3-HE_{12}-SNAs, HE_{12}-SNAs, or Cy3-ssDNA (1 μM) at 37°C for 24 hours. Subsequently, cells were fixed with 2% paraformaldehyde/1X PBS. Cells were then washed with 1X PBS and mounted with Prolong Gold (Invitrogen, USA) and cured overnight at 4°C. Images were recorded using a Leica LS Microscope (Leica, Germany) and images were analyzed using the LASX software (Lecia, Germany).

Cellular uptake of Nile Red-loaded nanoparticles

Encapsulation of Nile Red

Nile Red was prepared as a 10 mM working solution in acetone. Loading of the structures was achieved by adding 20 μL of Nile Red to a glass vial, followed by solvent evaporation in open air to achieve a thin drug film. HE_{12}-DNA conjugates in water were then added the drug film, mixed and followed by the addition of the assembly buffer (final solution: 100 μL at 10 mM in 1xTAMg buffer) with excess Nile Red (2 mM). The mixture was vortexed heavily to allow re-suspension of the drug molecules, and was annealed overnight (95°C-4°C over 4 hours). The mixture was purified by preparative centrifugation (15,000 x g, 4°C, 1 hour) between 2-4 times) and the concentration of encapsulated Nile Red was determined by fluorescence spectroscopy. Fluorescence emission spectra of each sample were collected in triplicate by mixing a 25 μL aliquot of the purified sample with 75 μL of acetone and recording the emission spectra (Nile Red:

exc. 535nm) of the sample in a microplate reader. A standard curve for [dye] versus maximal fluorescence intensity was used to calculate the [dye] present in each sample.

Flow cytometry experiments

HeLa cells were seeded at a density of 5 x 10^5 in a 6 well plate. After 24 hours, the cells were incubated with HE_{12}-SNAs + Nile Red or Nile Red alone (125 µL of sample added in a total media volume of 1 mL). The final concentration of Nile Red was 375 nM in both samples. After 12 hours of incubation, cells were detached, washed and resuspended in 1x PBS, followed by fixing with 2% paraformaldehyde. Samples were then processed using FACS FORTESSA. All measurements were performed in triplicates for error analysis.

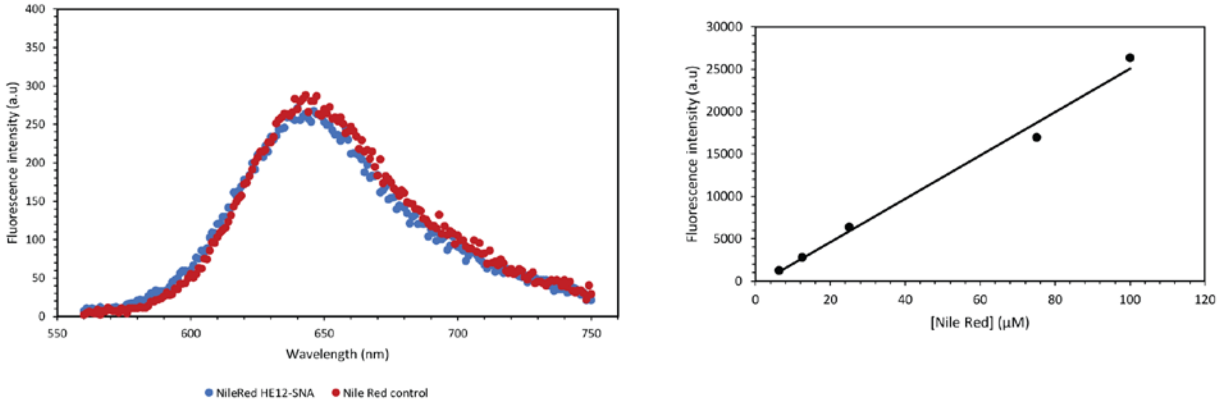

Supporting Figure 21. Quantification of encapsulated Nile Red in HE_{12}-SNAs and Nile Red control (acetone:water 3:1 v/v) by fluorescence spectroscopy measurements prior to flow cytometry studies. Following purification of un-encapsulated Nile Red, the initial concentration of dye was prepared at [Nile Red] = 3 µM in both HE_{12}-SNA and Nile Red control, to yield a final dye concentration of 375 nM in cell media.

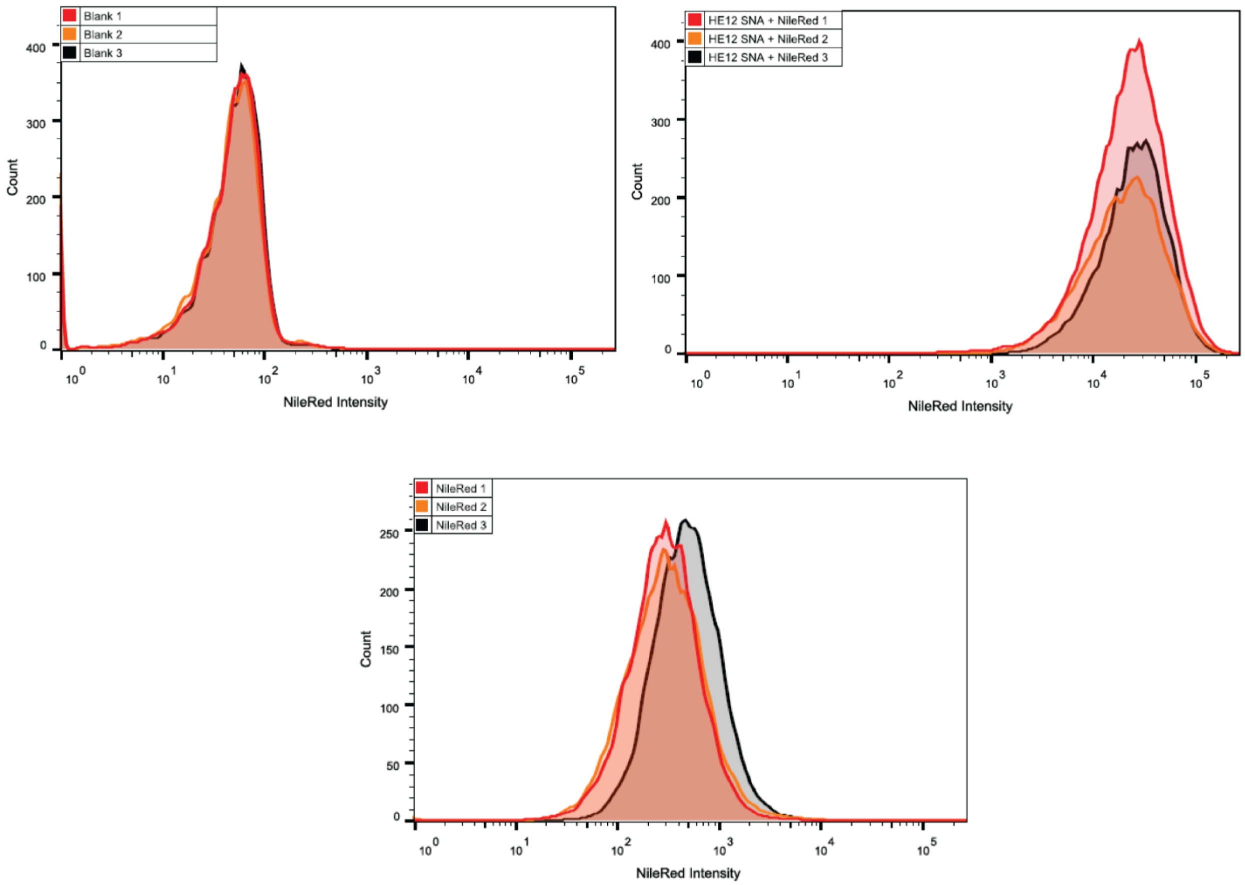

Supporting Figure 22. Flow cytometry measurements showing the increased uptake of Nile Red when delivered by HE_{12}-SNAs. Final [Nile Red] = 375 nM in cell culture media. Nile Red images were acquired at exc. wavelength 535 nm, emission 670 nm. These images were used for quantification of Nile Red uptake. The studies were performed in triplicates.

Fluorescence Microscopy for Nile Red Encapsulation

HeLa (adenocarcinoma) cells were seeded at a density of 5 x 10^5 in 8-well slides. After 24 hours, cells were incubated with HE_{12}-SNAs, HE_{12}-SNAs + Nile Red, or Nile Red alone (50 µL of sample added in a total media volume of 250 µL, final dye concentration = 400 nM) at 37°C for 2 hours. Subsequently, cells were fixed with 2% paraformaldehyde/1X PBS. Cells were then washed with 1X PBS and mounted with Prolong Gold (Invitrogen, USA) and cured overnight at 4°C. Images were recorded using a Zeiss AxioImager and images were analyzed using the Zen software (Zeiss, USA).

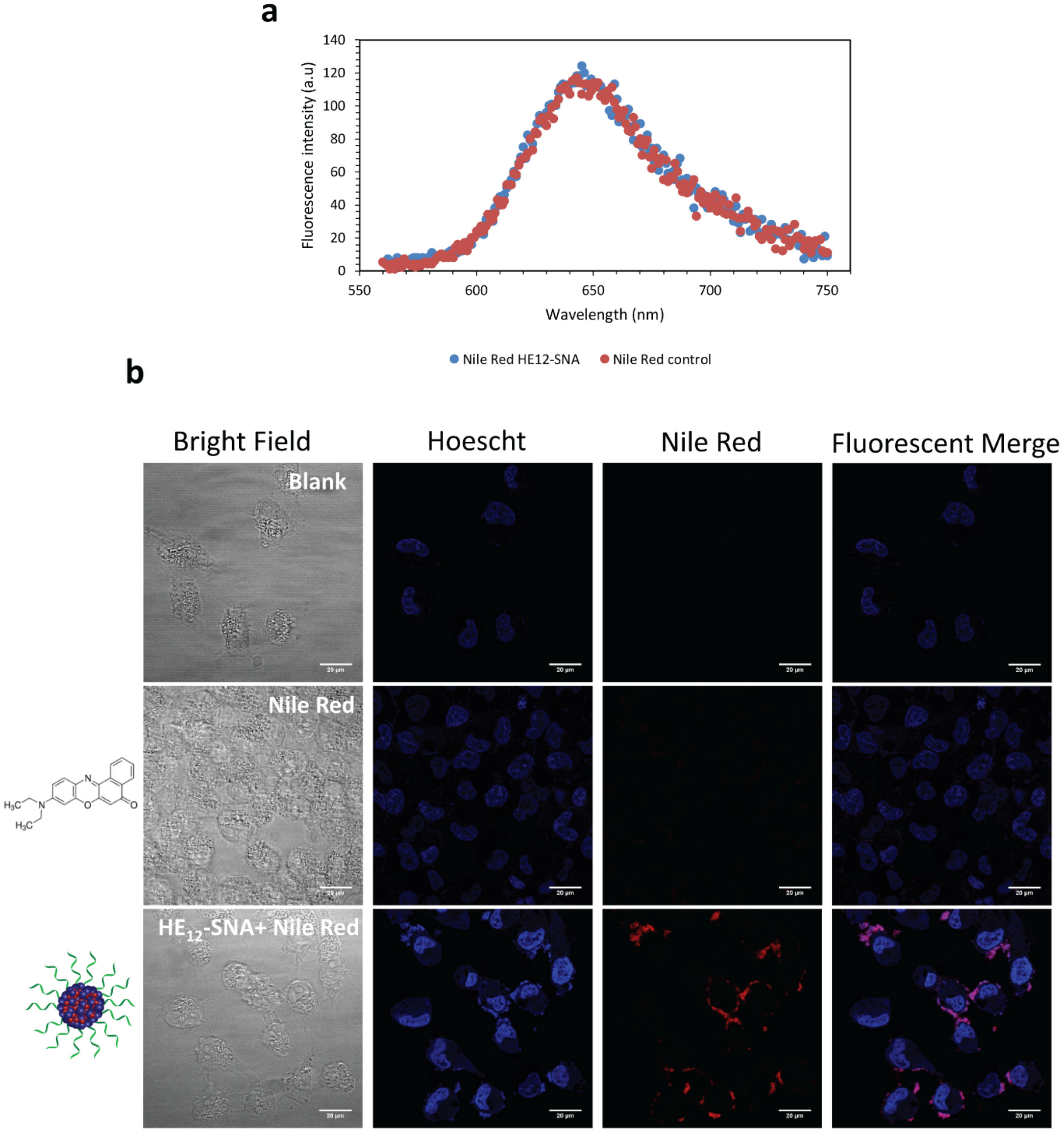

Supporting Figure 23. Fluorescence data showing Nile Red internalization in HeLa cells. a) Quantification of encapsulated Nile Red in HE_{12}-SNAs and Nile Red control by fluorescence spectroscopy measurements prior to confocal microscopy studies. Following purification of excess un-encapsulated Nile Red, the initial concentration of dye was prepared at [Nile Red] = 2 μM in both HE_{12}-SNA and Nile Red control, to yield a final dye concentration of 400 nM in cell media. b) Confocal fluorescence microscopy images demonstrating the cellular uptake of Nile Red when in HeLa cells. All samples were incubated for 12 hours with Nile Red or HE_{12}-SNA + Nile Red. Nile Red images were acquired using Ar Ion laser 514 nm and Hoescht 3342 was used as a nuclear stain.

XII. *In vitro* cell studies

MTS Assay for BKM120 and DOX-BKM120 Cell Viability

Cell viability of HeLa cells after BKM120 treatments or in combination with Doxorubicin, was measured using the Cell-Titer Blue assay (Promega, USA). Briefly, cells were seeded in 96-well plates at a density of $1x10^5$. After 24 hours, BKM120 was added (final concentrations: 0.06, 0.12, 0.3, 1.52, 7.6 µM) for single treatments. When BKM120 was added in combination with Doxorubicin, BKM120 was varied between 0.12, 1.2, and 12 µM, while Doxorubicin was maintained at 0.1, 1, and 10 µM for each of those experiments. Subsequently after 24 hours, plates were analyzed at using a Bio Plater Reader using 560 nm Ex/590 nm emission. Data was plotted and analyzed using the GraphPad Prism Software.

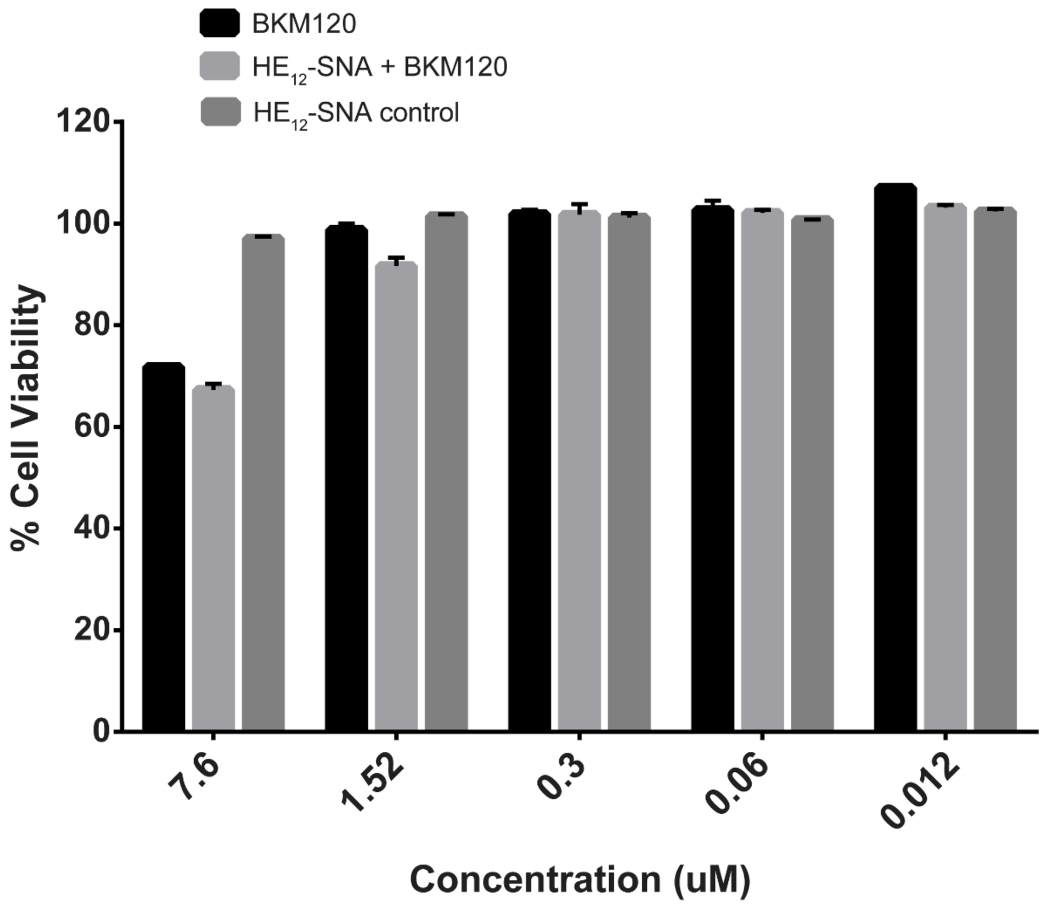

Supporting Figure 24. *In vitro* cytotoxicity of BKM120-loaded HE_{12}-SNAs in HeLa cells.

Apoptosis studies in primary CLL patient cells

Primary B-CLL lymphocytes were maintained in RPMI complemented with 10% fetal bovine serum (FBS). BMS2 stromal cell line were maintained in Dulbecco's modified eagle medium (DMEM) supplemented with 10% FBS. BMS2 cells were plated at $70x10^4$ cells/ml in 24-well plates before cocultured with primary B-CLL lymphocytes and incubated at 37ºC, 5% CO_2. For apoptosis assays, $3x10^6$ B-CLL lymphocytes were plated in the presence or absence of stromal cell (BMS2) and incubated for 1 hour at 37ºC, 5% CO_2. Cells were then treated with vehicle, nanoparticle, BKM120, and BKM120-loaded nanoparticles for 24 and 48 hours.

AnnexinV /propidium iodide analysis

Cells were harvested, washed with PBS then incubated with 1 μL Annexin V APC conjugated plus 0.5 μg/ml propidium iodide in 100 μL binding buffer for 15 min at room temperature. Cells were then analyzed with a FACSCalibur flow cytometer.

Cleaved caspase-3 analysis

Cells were harvested, washed with PBS then fixed for 10 min with 1% paraformaldehyde. After washing, cells were permeabilized and non-specific sites blocked in PBS containing 3% FBS and 0.01% triton X100. Cells were then incubated for 1 hour with an anti-caspase-3 FITC conjugated antibody then analyzed with a FACSCalibur flow cytometer.

Immunostimulation TNF-α ELISA Assays

Immunostimulation assays were performed using RAW264.7 cells (ATCC), mouse monocytes. Briefly, cells were seeded in a 96-well plates at a density of $1x10^5$. Subsequently, after 24 hours, HE_{12}-SNAs (2 μM), ssDNA (2μM), LPS 500 ng/mL (lipopolysaccharide), poly IC 10 ug/mL+Transfection, were added to the cells and incubated for 5, 24, and 48 hours. At each time-point, supernatants were collected and frozen at -20°C. When all samples were collected, a TNF-α ELISA assay was performed according to the manufacturer's instructions (eBioscience) using the Instant ELISA TNF-α kit. Results were measured using a Bio plate reader at a wavelength of 630 nm and data was analyzed using the Graphpad Prism Software.

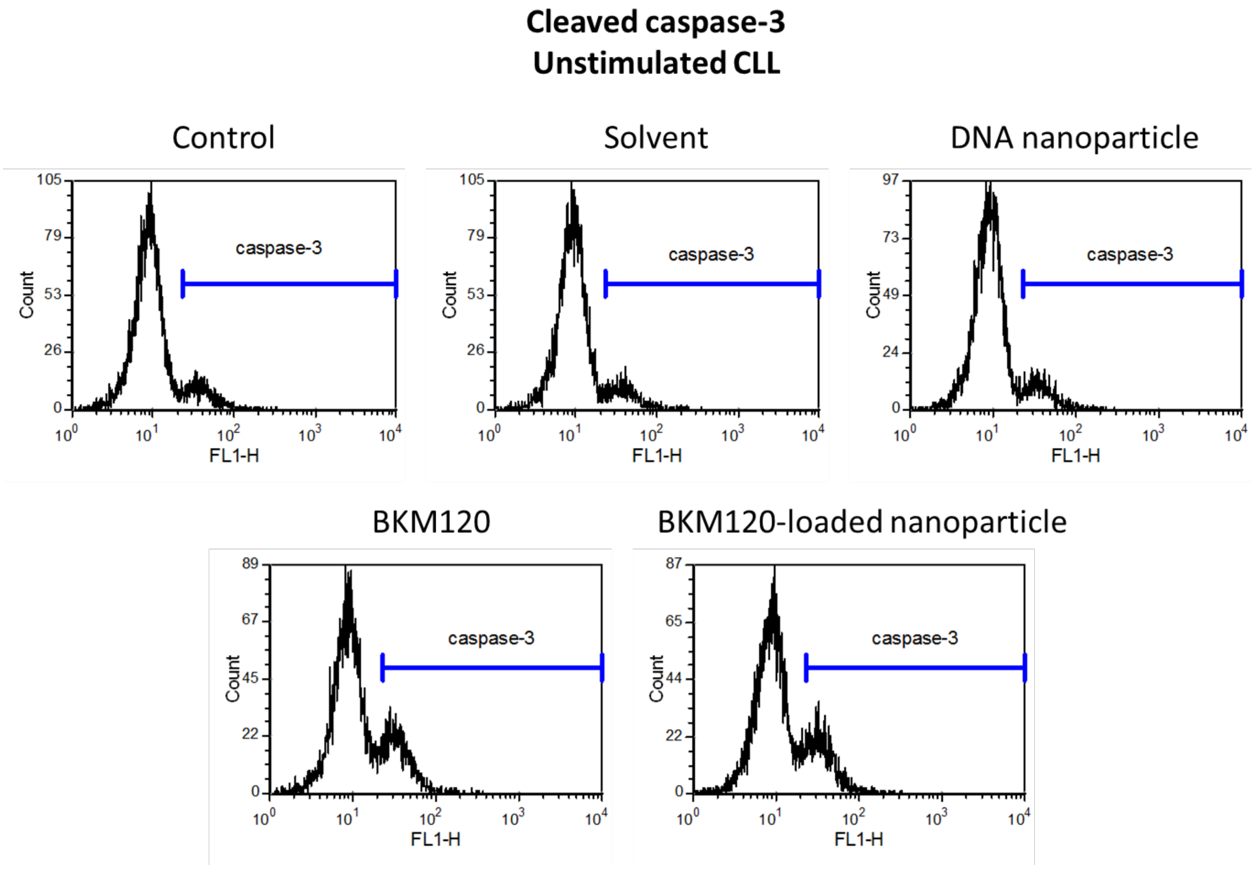

Supporting Figure 25. Flow cytometry data showing the amount of cleaved-caspase 3 in unstimulated primary CLL lymphocytes.

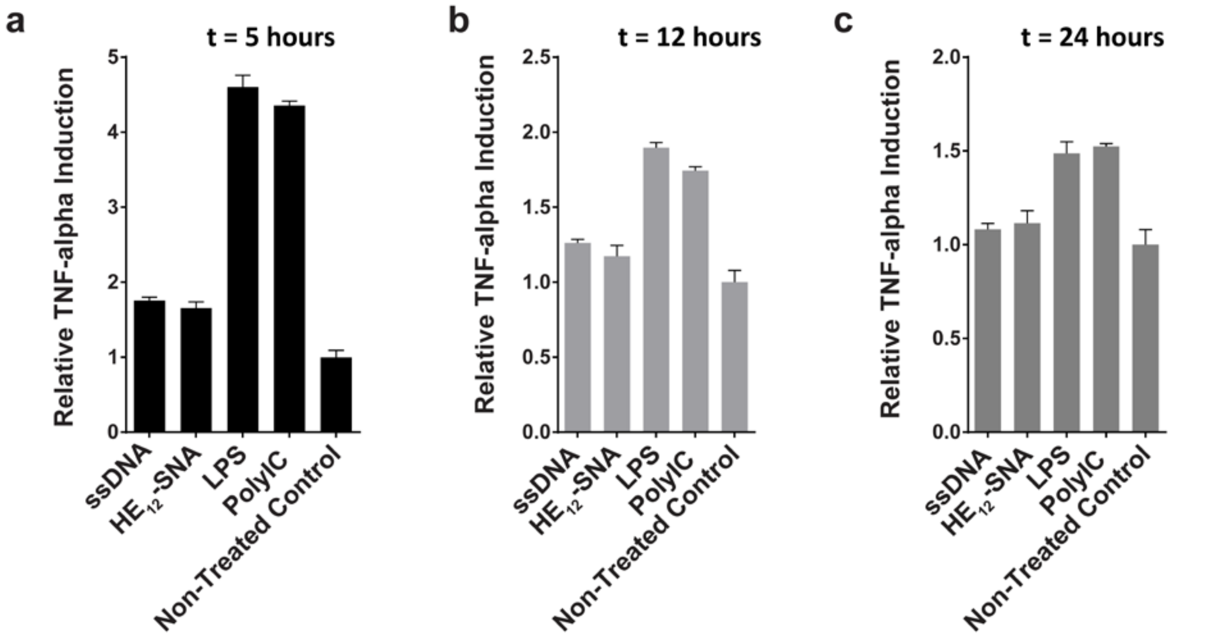

Supporting Figure 26. Time-dependent effect of HE_{12}-SNAs on TNF-alpha induction by RAW264.7 mouse monocytes, measured at a) 5 hours, b) 12 hours and c) 24 hours after treatment.

XIII. HSA binding studies:

HE_{12}-SNAs were prepared as a 10 µM solution by thermal annealing 95°C-4°C over 4 hours. For binding studies, 1 µL of HSA stock (526 µM) was added to 10 µL of DNA nanoparticles and incubated for 2 hour prior to analysis by agarose gel electrophoresis. For control experiments, 10 µL of HE_{12}-DNA micellar structures were denatured by the addition of denaturing solution of urea (10 µL of each of 8 M urea) and depletion of magnesium cations prior to HSA addition in an EDTA containing TBE buffer, exposing their long aliphatic chains. Titrations of human serum albumin (HSA) prepared at 526 µM were added to the denatured HE_{12}-SNA solution to yield final HSA dilutions of 1/1000, 1/100, 1/50, 1/10, 1/5, 1/2 and undiluted excess HSA (526µM stock). The samples were analyzed under denaturing 20% PAGE.

As another control, DNA nanoparticles were incubated with 10% FBS for different time points, and subsequently denatured with 2x formamide (without the addition of proteinase K enzyme). The structures were then run under 20% denaturing PAGE.

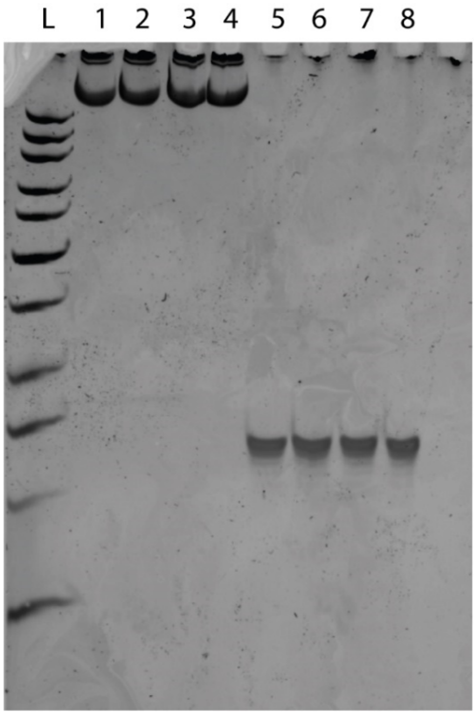

Supporting Figure 27. PAGE analysis of denatured HE_{12}-DNA conjugate strands incubated with 10% FBS. Incubation times: 0 mins, 1 hour, 2 hours, 4 hours. Lanes 1-4 are in the presence of 10% FBS, lanes 5-8 in the absence of FBS.

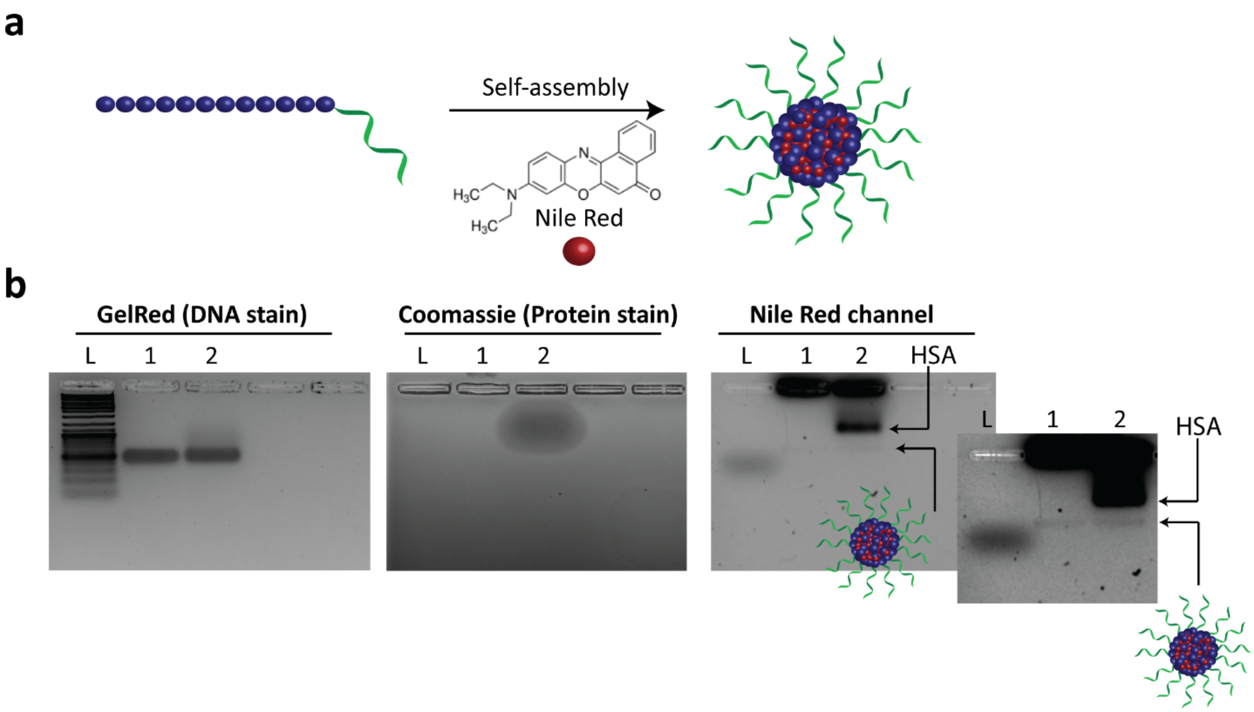

Supporting Figure 28. HE_{12}-SNA binding studies with human serum albumin (HSA). a) Preparation of Nile Red-loaded nanoparticles. b) Agarose gel electrophoresis of HE_{12}-SNAs prior (Lane 1) and post incubation (Lane 2) with HSA. The gels were visualized under Gel Red DNA stain channel (left panel), Commassie Blue protein stain (middle panel) and Nile Red channel (right panel).

The effect of HSA on release of encapsulated drug was investigated. This would provide some indication as to how our drug-loaded structures would behave *in vivo*. Our studies were conducted by initially preparing Nile Red-loaded DNA nanoparticles (Figure SF 22). These structures would allow to study both: the release of Nile Red upon HSA addition, and the direct interaction of DNA nanoparticles with HSA. Following HSA addition (5x molar excess) and incubation for 2 hours, the products were analyzed by agarose gel electrophoresis AGE. As illustrated in Figure SF22, upon incubation, no interaction is observed between the DNA nanoparticles and HSA protein. These results are further confirmed in the Nile Red channel, where two distinct populations of DNA particles and HSA are observed. The analysis of AGE data was complicated because under the gel electrophoretic conditions, the dye molecules diffused out of the DNA particles, and remained in the gel wells. Thus, it was difficult to use AGE data to determine Nile Red release. If we assume that the non-penetrating that is not associated with HSA

represents Nile Red originally encapsulated in the nanoparticle, then the data would suggest that only a small fraction of Nile Red is released upon HSA addition, and was bound to protein, possibly to the HSA hydrophobic binding pockets.

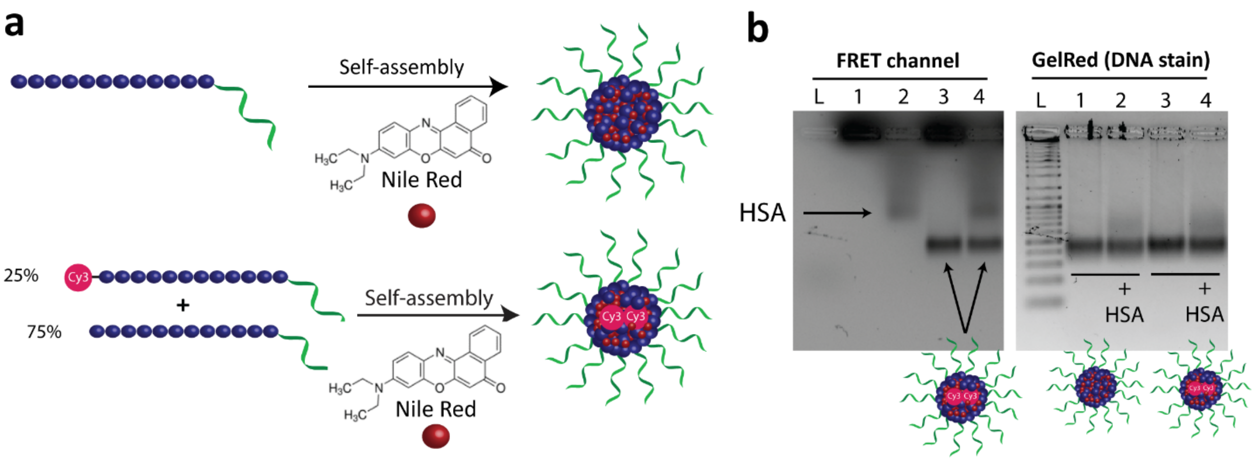

Supporting Figure 29. HSA binding studies of HE$_{12}$-SNAs and the effect on encapsulated cargo. a) Generation of Nile Red-loaded unlabeled and Cy3-labeled DNA nanoparticles. b) AGE of Nile Red-loaded cyanine-labeled nanoparticles incubated with HSA protein visualized under FRET channel (excitation 546 nm, emission 650 nm) and GelRed DNA stain. Lanes 1 &2: unlabeled DNA nanoparticles, Lanes 3&4: Cy3-labeled nanoparticles, pre-and post HSA incubation.

To further investigate the interaction with HSA, we designed a Nile Red-loaded Cy3-DNA nanoparticle system (Figure SF29). Due to the spectral overlap between Cy3 and Nile Red, simultaneous observation of released Nile Red molecules bound to HSA and the labeled DNA nanoparticles is possible under one fluorescence channel. In other experiments, we found that under gel electrophoretic conditions Nile Red molecules diffused out of the nanoparticle core, and a small portion of the dye molecules was found associated with HSA (Figure SF29). These findings would hence allow us to track the mobility shift of the HSA protein by monitoring Nile Red fluorescence. Thus, the spectral overlap between Nile Red and Cy3 would then allow direct observation of HSA and Cy3 nanoparticles under a detection channel common for both Nile Red and Cy3. As illustrated in Figure SF23, the lack of interaction between labeled DNA particles and HSA is demonstrated through the presence of two populations with different mobility shifts. Additionally, the amount of DNA was observed to remain mostly unchanged after HSA incubation (GelRed channel).

XIV. *In vivo* studies:

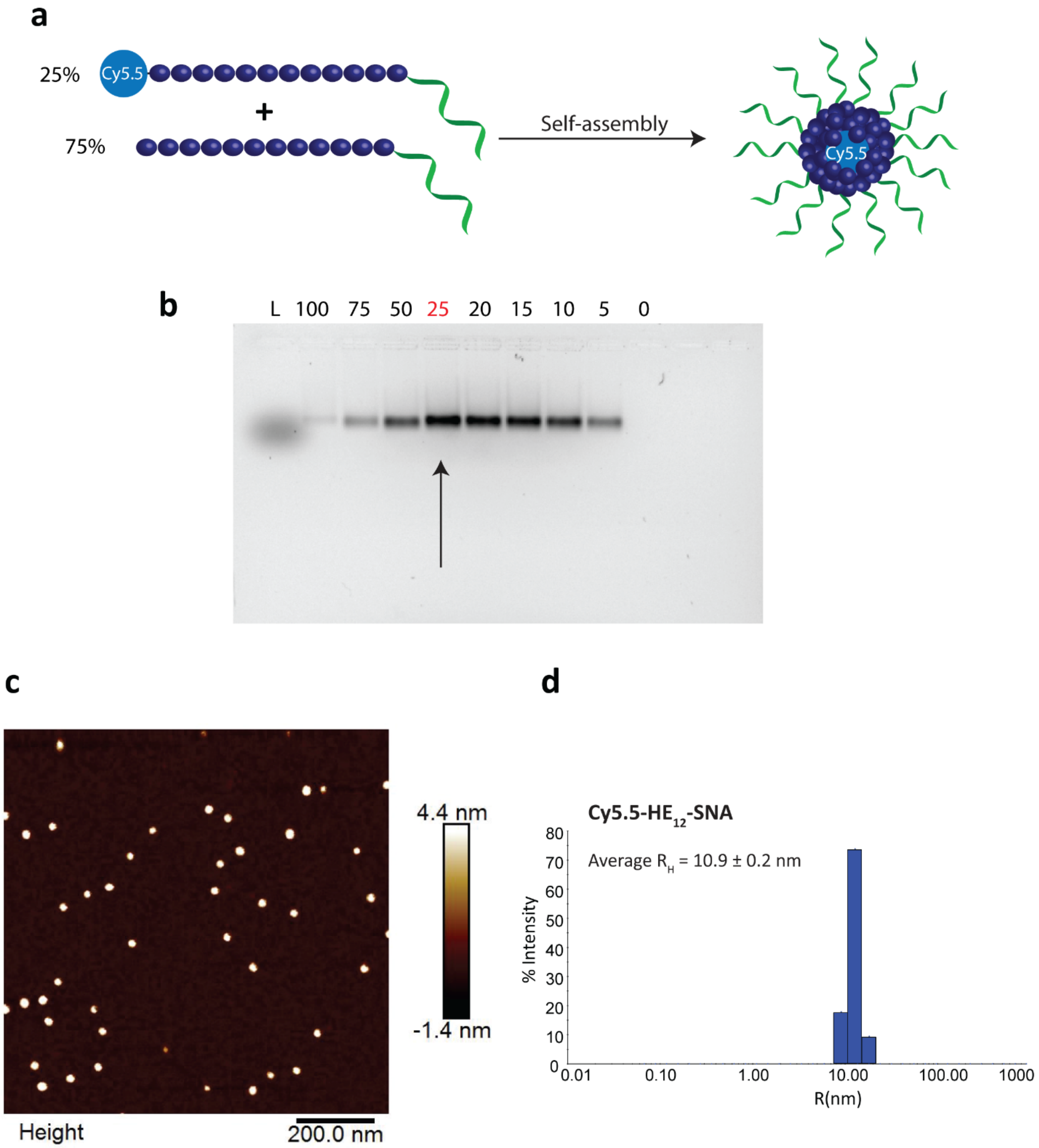

Supporting Figure 30. Characterization of Cy5.5-labeled HE_{12}-SNAs for *in vivo* biodistribution studies. a) General methodology for preparing Cy5.5-labeled DNA nanoparticles. b) Agarose gel electrophoresis (AGE) characterization of Cy5.5-labeled particles imaged under Cy5.5 channel. Numbers on lanes indicate the percentage of labeled strands in the final structure. At ratio 25:75 of Cy5.5-HE_{12}-DNA/unlabeled HE_{12}-DNA strands, the structures show highest fluorescence

intensity and morphological integrity. c) Atomic force microscopy (AFM) images of Cy5.5-labeled nanoparticles showing monodisperse structures with average diameter of 26.8 ± 2.7 nm. c) DLS histogram of Cy5.5-labeled HE_{12}-SNAs showing an in-solution hydrodynamic radius of 11.8 ± 0.4 nm.

Preparation of Cy5.5-labeled nanoparticles

Cy5.5 phosphoramidite was appended from the 5' of the HE_{12}-DNA through attachment to one end of the polymer chain (opposite to the DNA) to yield Cy5.5-HE_{12}-DNA conjugates. Cy5.5-labeled DNA nanoparticles were prepared by mixing Cy5.5-labeled HE_{12}-DNA with unlabeled HE_{12}-DNA conjugates in a 25:75 molar ratio (Total DNA concentration 17 µM, total Cy5.5-DNA concentration 4.25 µM, total volume 100 µL). This ratio of Cy5.5 labeled/unlabeled strands resulted in high fluorescence intensity of the dye molecules and clean assemblies. For *in vivo* studies, additional UV-Vis measurements were conducted to ensure similar dye absorbance of the Cy5.5-labeled nanoparticles to Cy5.5-ssDNA.

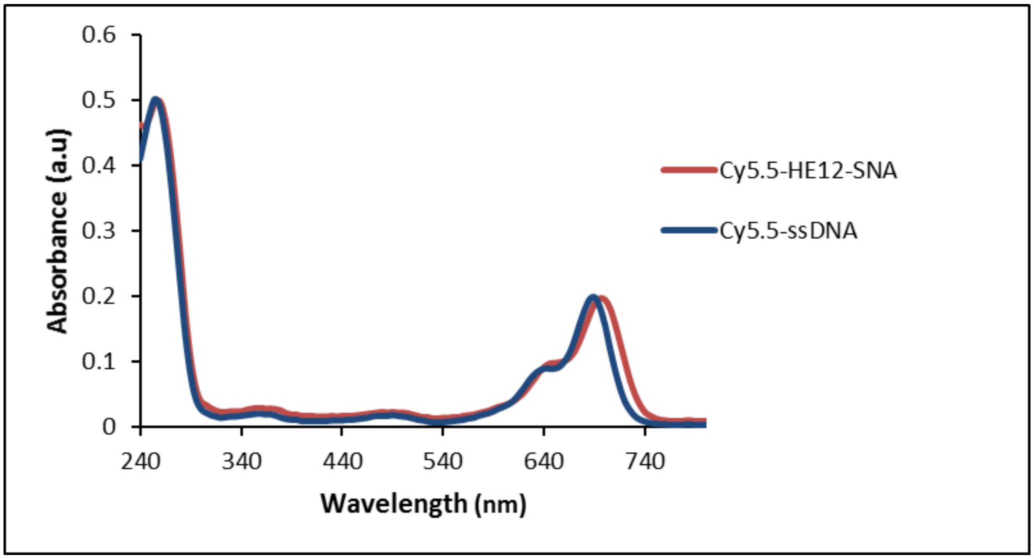

Supporting Figure 31. UV-Vis spectroscopy of Cy5.5-labeled nanoparticles and Cy5.5-ssDNA used for *in vivo* studies.

In vivo biodistribution in CD1 mice

Experiments for biodistribution were performed according to a protocol approved by McGill University Animal Care Committee and Lady David Institute for Medical Research Animal Facility (approval # 2013-7350). 12 CD-1 mice will be used to determine the biodistribution of Cy5.5-labeled HE_{12}-SNAs by using the IVIS system. Mice were divided in 4 groups: control (4 mice), unlabeled HE_{12}-SNAs (2 mice), Cy5.5 (2 mice), Cy5.5-ssDNA (2 mice) and Cy5.5-labeled HE_{12}-SNAs. The biodistribution was evaluated after intraperitoneal or intravenous route of administration. HE_{12}-DNA nanoparticles (100 μL at 17 μM total DNA) were injected in a single dose. The mice were then anesthetized with isoflurane, and then the fluorescence measured using the *In Vivo* Imaging System (IVIS), 15 mins, 30 mins, 1 hours, 2 hours, 4 hours, 6 hours and 24 hours after treatment. Survival time: 30 hours. Euthanasia: After 30 hours, the mice will be euthanized with isoflurane followed by cervical dislocation.

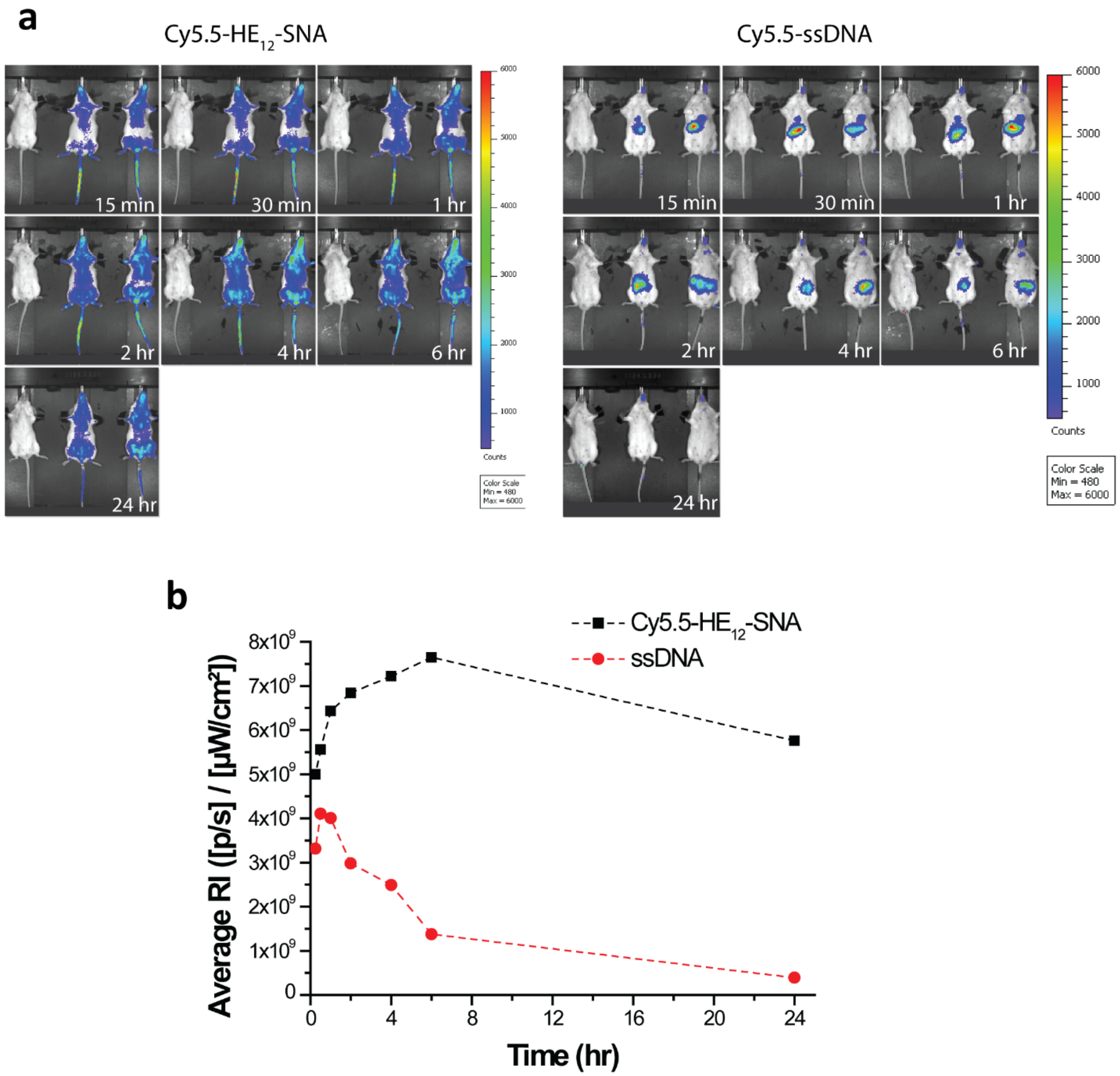

Supporting Figure 32. *In vivo* biodistribution of Cy5.5-labeled HE_{12}-DNA nanoparticles after intravenous administration. a) Cy5.5 fluorescence data overlaid on X-ray images measured over time. b) Quantified biodistribution data of Cy5.5 intensity measured as a function of time for Cy5.5-HE_{12}-DNA nanoparticles.

Biodistribution in HCT116 xenograft models

Intraperitoneal injection

Six female nude mice received subcutaneously injection in the left flank with $3x10^6$ HCT116 cells in 0.2 mL of saline through a 27-gauge needle. Three weeks later, when the tumor reached a mean tumor volume of 140 mm3, the 5 mice bearing subcutaneous tumor were divided in control (2 mice) or test groups (3 mice). Mice from the test group received intraperitoneal injection of Cy5.5-conjugated DNA nanoparticles in a single dose (100 µL at 17µM total DNA). To assess the fluorescence bio-distribution, mice were anesthetized with isoflurane then fluorescence was imaged 0 minutes, 15 minutes, 30 minutes, 1 hour, 2 hours, 4 hours, 6 hours, and 24 hours after injection of Cy5.5-conjugated DNA nanoparticles using the In Vivo Imaging System (IVIS).

Intravenous injection

Six female nude mice received subcutaneously injection in the left flank with $3x10^6$ HCT116 cells in 0.2 mL of saline through a 27-gauge needle. Three weeks later, when the tumor reached a mean tumor volume of 140 mm^3, the 6 mice bearing subcutaneous tumor were divided in control (2 mice) or test groups (3 mice). Mice from the test group received intravenous injection of Cy5.5-conjugated DNA nanoparticles in a single dose (300 µL at 17µM total DNA). To assess the biodistribution, mice were anesthetized with isoflurane then fluorescence was imaged 0 minutes, 15 minutes, 30 minutes, 1 hour, 2 hours, 4 hours, 6 hours, and 24 hours after injection of Cy5.5-conjugated DNA nanoparticles using the In Vivo Imaging System (IVIS).

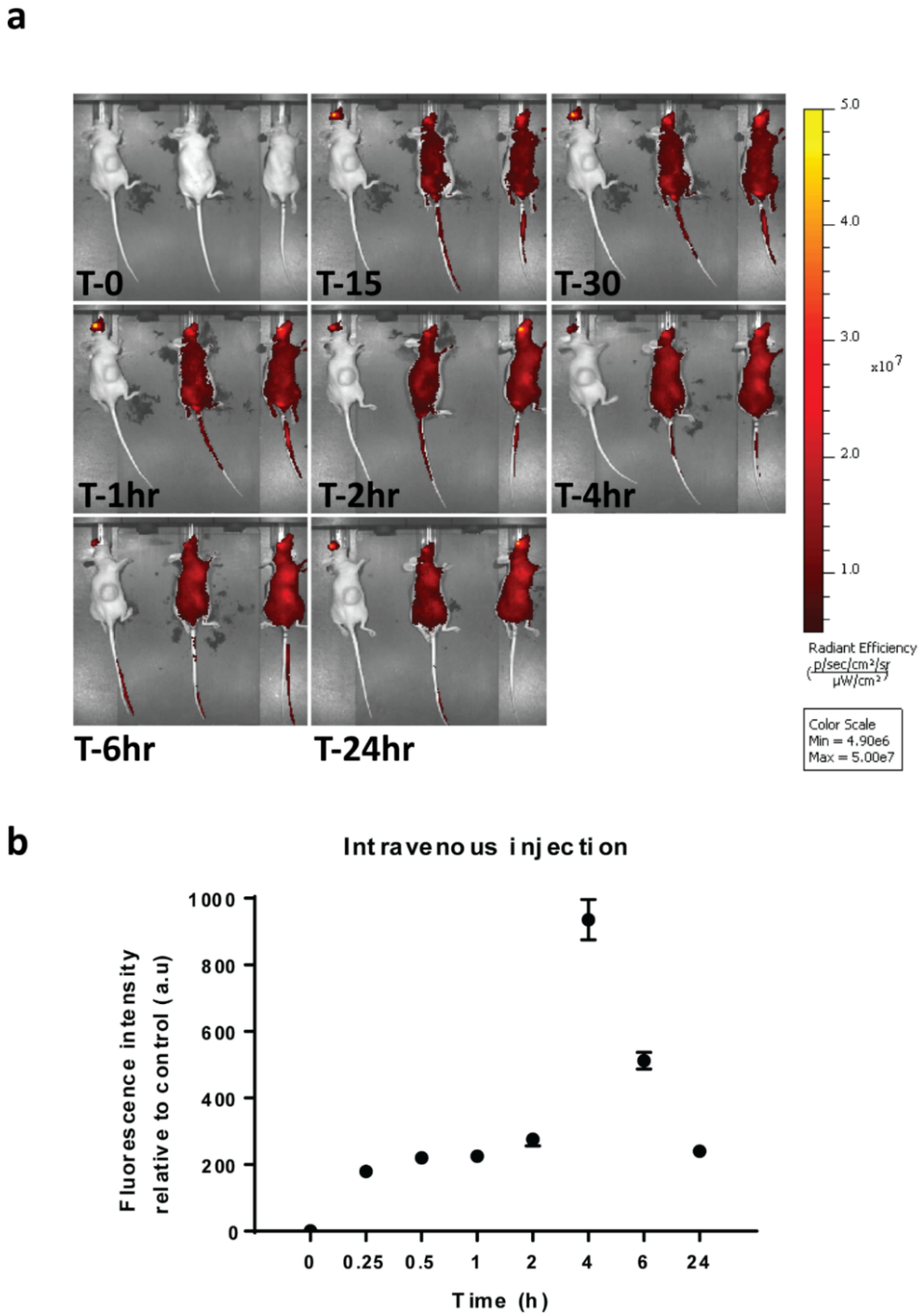

Supporting Figure 33. Biodistribution of Cy5.5-labeled HE_{12}-SNAs in HCT116 xenografts following intravenous injection.

XV. References

1. S. L. Beaucage, R. P. Iyer. Tetrahedron **1992**, 48, 2223

2. T. G. Edwardson, K. M. Carneiro, C. J. Serpell, H. F. Sleiman. *Angew. Chem. Int. Ed. Engl.* **2014,** *53* (18), 4567-71.

3. P. Greenspan, E. P. Mayer and S. D. Fowler, *J. Cell Biol.*, 1985, **100**, 965-973.

4. J. W. Conway, C. K. McLaughlin, K. J. Castor, H Sleiman. *Chem. Comm.* **2013**, *49*, 1172.

5. J. J. Fakhoury, T. G. Edwardson, J. W. Conway, T. Trinh, F. Khan, M. Barlog, H. S. Bazzi, H. F. Sleiman. *Nanoscale* **2015,** *7* (48), 20625-34.
